# Highly Concentrated Peroxodicarbonate for Efficient Oxidative Degradation of Kraft Lignin

**DOI:** 10.1002/cssc.202500741

**Published:** 2025-06-09

**Authors:** Niclas Schupp, Theresa Rücker, Elisabeth Glöckner, Bernd Wittgens, Siegfried. R. Waldvogel

**Affiliations:** ^1^ Department of Electrosynthesis Max Planck Institute for Chemical Energy Conversion Stiftstrasse 34‐36 45470 Mülheim an der Ruhr Germany; ^2^ Department of Process Technology SINTEF Industry Richard Birkelands vei 3 7034 Trondheim Norway; ^3^ Karlsruhe Institute of Technology (KIT) Institute of Biological and Chemical Systems‐Functional Molecular Systems (IBCS‐FMS) Kaiserstraße 12 76131 Karlsruhe Germany

**Keywords:** electrolysis, lignins, NMR spectroscopy, oxidations, peroxodicarbonates

## Abstract

The oxidative degradation of technically relevant types of Kraft lignin is efficiently accomplished by a significantly improved two‐step protocol. The key is the use of highly concentrated peroxodicarbonate solution which selectively oxidizes the lignin particles at moderate temperature which are thermally treated in a subsequent step. The liberation of low‐molecular‐weight phenols occurs when the oxidizer is already consumed enhancing the yield of target compounds strongly up to 15.6 wt%. Co‐generated carbonates in this transformation represent the make‐up chemical of pulping plants. This makes this approach suitable and attractive to be implemented as a bolt‐on or integrated into the Kraft pulping process. The green metrics clearly indicate the sustainable and superior features of the method established.

## Graphical abstract

A two‐step protocol is developed for the efficient oxidative degradation of Kraft lignin. This method utilizes a highly concentrated peroxodicarbonate solution at moderate temperatures, followed by thermal treatment, significantly enhancing monoaromatic yield up to 15.6 wt%. Cogenerated carbonates in this transformation represent the make‐up chemical of pulping plants.

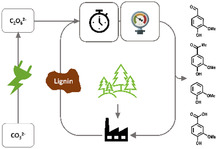

## 1 Introduction

In times of a global climate and energy crisis, it is important to overcome these given challenges and develop novel and sustainable technologies and mindsets.^[^ ^1^, ^2^, ^3^, ^4^ ^]^ One of the greatest bottlenecks is the transformation from fossil carbon sources‐based chemical processes into green alternatives. The exploitation of renewable feedstock is therefore receiving growing attention. Lignin is becoming increasingly prevalent as is the second‐most abundant biopolymer.^[^ ^5^, ^6^, ^7^ ^]^ It is a residual material from the pulping industry and can be used to produce a variety on phenolic monomers.^[^ ^5^, ^6^, ^7^, ^8^ ^]^ Lignin is accessible in strongly different chemical features due to the various existing pulping processes causing chemical modifications of the native lignin structure. It is considered as an underutilized side‐stream which is incinerated to generate process heat and recover inorganic process chemicals. However, only 2% of lignin is commonly used as a feedstock for the manufacturing of materials or high‐value‐added products.^[^ ^9^ ^]^ The feasibility of a complete, selective depolymerization of lignin into one type of monomers has been recently challenged in a proposed “liquefy first” approach in order to emphasize the heterogeneous nature of lignin and resulting yields.^[^ ^10^ ^]^ The most common available and least expensive lignin type is Kraft lignin.^[^ ^6^, ^11^, ^12^ ^]^ Unfortunately, the alkaline Kraft process results in strongly chemically modified lignin which makes degradation extremely challenging.^[^ ^11^, ^13^ ^]^ In the past decades, different methods to degrade technically relevant lignin have been reported.^[^ ^14^, ^15^, ^16^, ^17^, ^18^, ^19^, ^20^ ^]^

**Scheme 1:**
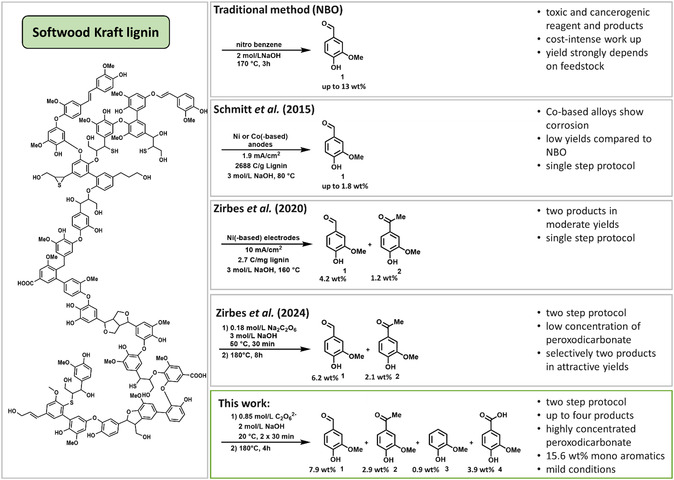
Selected strategies for oxidative degradation of Kraft lignin into monoaromatics. Softwood Kraft lignin structure redrawn from Crestini et al. and modified.^[^ ^11^, ^12^ ^]^

Among those published were various two‐stage protocols for the degradation of Kraft lignin.^[^ ^21^, ^22^ ^]^ Despite the toxic byproducts of the degradation of Kraft lignin by nitrobenzene oxidation, it is considered as the gold standard to determine the maximum yield of vanillin achievable (**Scheme** **1**).^[^ ^23^, ^24^ ^]^ The use of chemical oxidizers leads to reagent waste that has to be treated. Electric current is regarded as a promising alternative. It is inexpensive, inherently safe, and reduces the amount of harmful, toxic waste.^[^ ^25^, ^26^, ^27^, ^28^ ^]^ The direct electrochemical degradation of various technically relevant lignin‐types has been reported.^[^ ^20^, ^29^, ^30^, ^31^, ^32^, ^33^, ^34^, ^35^ ^]^ The use of active nickel anodes provided access to selective formation of vanillin. However, the yields were in the range of 1%–2% leading to an efficient isolation by ion exchange resins without neutralization of the reaction mixture.^[^ ^36^, ^37^ ^]^ Further advancement was achieved by a high‐temperature protocol with nickel anodes, affording yields of up to 4.2 wt% of vanillin (**1**).^[^ ^34^ ^]^ This concept was successfully adopted to different types of organosolv lignin.^[^ ^33^ ^]^ Within the past decades, boron‐doped diamond (BDD) electrodes have become commercially accessible for electrosynthesis.^[^ ^38^, ^39^ ^]^ These ultrastable anodic materials allow the efficient use for the ex‐cell electrolysis of platform oxidizers.^[^ ^40^, ^41^, ^42^, ^43^ ^]^

As such, peroxodicarbonate, for example, has considerable potential for large‐scale industrial processes, as it can be produced as an alternative anodic reaction for oxygen evolution as part of electrochemical water splitting.^[^ ^44^ ^]^

This is of great interest if intermittent electricity is to be exploited. A well‐described approach is the production of sodium peroxodicarbonate from an aqueous solution.^[^ ^45^, ^46^, ^47^, ^48^, ^49^ ^]^ Peroxodicarbonate can be understood as the dimer of CO_3_^2−^ connected via a peroxide bond and differs strongly in its behavior to H_2_O_2_, however, peroxodicarbonate is only accessible electrochemically.^[^ ^48^, ^50^, ^51^, ^52^ ^]^

By exploiting the unique properties of peroxodicarbonate, Zirbes et al. developed a protocol to degrade Kraft lignin into vanillin (**1**) with yields of up 6.2 wt%.^[^ ^52^ ^]^ This protocol was also used in industrial approaches.^[^ ^53^, ^54^ ^]^ However, the approach was limited by concentrations of peroxodicarbonate of 0.28 mol L^−1^, which led to a huge amount of electrolyzed carbonate needed in order to keep a certain ratio between oxidizer and lignin.

**Scheme 2:**
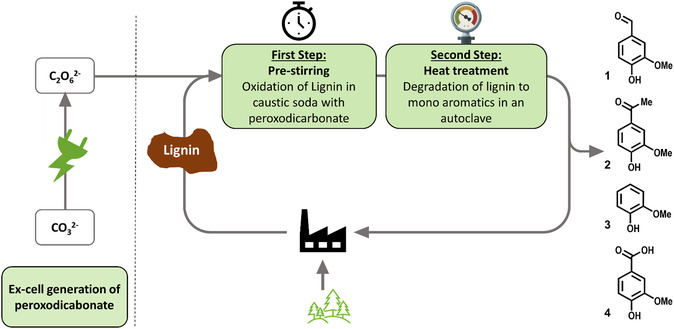
Schematic representation of lignin degradation. First, peroxodicarbonate is produced electrochemically in an ex‐cell approach. In a first step, lignin is prestirred with peroxodicarbonate in caustic soda. In the second final step, the heat treatment is performed.

These findings provide a promising approach for the use of highly concentrated peroxodicarbonate to degrade lignin affording even higher yields of **1** as well as other value‐added monoaromatics (**Scheme** **2**). This could enable more sustainable and cost‐efficient industrial production that results in less waste produced per ton of Kraft lignin.

To achieve high concentrations of peroxodicarbonate, heat dissipation is mandatory. Beyond standard lab setups, various customized flow cells for generating peroxodicarbonate have been documented.^[^ ^52^, ^55^ ^]^ All designs emphasize involving a cooling system, especially on the cathodic side. The first cell published to produce peroxodicarbonate utilizes a large BDD anode, making it costly. Despite integrated cooling, this setup only delivers low concentrations of peroxodicarbonate and requires high currents due to the large electrode surface.^[^ ^45^ ^]^ Concentrations exceeding 300 mmol L^−1^ were first achieved with the development of the cell by Seitz, which features a cooled copper casing with microcooling channels.^[^ ^48^ ^]^ Utilizing a small active anode surface, it reached high current density, enabling the production of peroxodicarbonate concentrations exceeding 900 mmol L^−1^. This high‐concentration peroxodicarbonate has demonstrated exceptional reactivity in various applications, outperforming lower‐concentration alternatives.^[^ ^48^, ^56^ ^]^ In this work, the utilization of highly concentrated peroxodicarbonate for Kraft lignin degradation is investigated. The protocol for highly concentrated peroxodicarbonate is optimized, and the effect on the yield of resulting monomers is examined.

## 2 Results and Discussion

**Figure 1:**
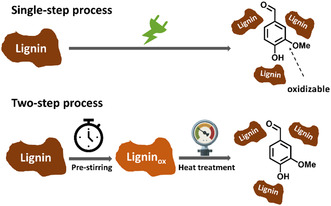
Conceptual presentation of the direct electrochemical singe‐step protocol (top) and the two‐step protocol (bottom).

First, experiments with lower concentrated peroxodicarbonate were performed and reproduced using the parameters published by Zirbes et al. (Scheme 1) for setting a benchmark for futher optimization of the degradation process and to

compare the effect of different concentrations of peroxodicarbonate.^[^ ^52^ ^]^ Therefore, the cell used by Zirbes et al. to generate peroxodicarbonate was used initially in this study. Kraft lignin Lineo, supplied by Stora Enso, served as technically relevant starting material. It should be emphasized that we used the two‐step protocol established by Zirbes et al. Peroxodicarbonate was produced electrochemically in an ex cell approach and subsequently intensively mixed (prestirred) with lignin. In this first step, the lignin is preoxidized but the target compounds 1–4 are not liberated. In a second, final step, the lignin was subjected to a strong heat treatment, in which the preoxidized lignin particle breaks up and the monomers are released (**Figure** **1**). In initial experiments, according to the parameters by Zirbes et al. the desired product 1 was formed with a 4.7 wt% yield and additionally the byproduct acetovanillone 2 with 1.7 wt%. With those conditions 73% of 1 compared to the reference protocol using nitrobenzene could be achieved (see Table S6, Supporting Information). Furthermore, the protocol for generation of peroxodicarbonate in high concentrations published by Seitz et al. was employed.^[^ ^48^ ^]^ A different autoclave was used for the thermal degradation step, being able to handle higher pressures up to 200 bar. The autoclaves employed by Zirbes et al. were limited to handling pressures of 8–10 bar, which proved inadequate for the pressures generated during the required heat treatment process. Following the protocol for high concentrated peroxodicarbonate (800 mmol L^−1^), this resulting solution was applied to the same type of Kraft lignin as in the former experiments to provide a clear comparison. Noteworthily, keeping the ratio of oxidizer to lignin constant, the volume of peroxodicarbonate solution used is lowered by factor 5 as a result of its fivefold higher concentration. The same yields of 1 as well as higher yields of up to 2.4 wt% of 2 were achieved. Additionally, 1.0 wt% of guaiacol 3 could be quantified. It is noteworthy that we could also afford at the same experiment high concentrations of 4. 4 wt% of vanillic acid **4**. This acid could be a result of overoxidation of the lignin treated and represents a compound of attractive value.^[^ ^57^, ^58^ ^]^ The formation of **4** is a strong indicator that high concentrations of peroxodicarbonate are more oxidation active and lead to a different ratio of monoaromatics. To investigate the possible overoxidation, the temperature of preoxidation (first step) was varied (**Table** **1**). By lowering the temperature of the pre‐oxidation step to 20 °C, the yields of 1 and 2 decreased. On the other hand, the yield of 4 as well as 3 increased.

**Table 1:** Oxidative degradation of Lineo Kraft lignin to mono aromatics vanillin (1), acetovanillone (2), guaiacol (3), and vanillic acid (4) using peroxodicarbonate as oxidizer at different prestirring temperatures.

Entry	Prestirring temperature (°C)	Yield **1** a) (wt%)	Yield **2** a) (wt%)	Yield **3** a) (wt%)	Yield **4** a) (wt%)	Total amount of monoaromatics (wt%)
1	70	4.1	1.9	1.2	2.3	9.5
2	60	5.1	2.4	1.4	2.6	11.5
3	50	4.7	2.4	1.0	4.4	12.5
4	40	3.9	2.1	1.1	4.9	12.0
5	30	3.1	1.9	1.3	5.0	13.3
6	20	2.7	1.6	1.5	4.9	10.7
7b)	20	2.6	1.5	1.0	5.3	10.4

a) The yield was determined by gaschromatography with flame ionization detector (GC‐FID) using *n–*dodecylbenzene as internal standard and refers to the amount of lignin employed.

b) Lignin was dissolved at 50 °C and then cooled to 20 °C before adding peroxodicarbonate. Reaction conditions: 100 mg Kraft lignin, 50 g 3 mol L^−1^ NaOH, 3 mmol peroxodicarbonate, c(Peroxodicarbonate) = 800 mol L^−1^, 30 min prestirring, heat treatment temperature: 180 °C, heat treatment time: 8 h.

A possible explanation for the increasing yields in vanillic acid could be the higher stability of peroxodicarbonate at lower temperatures. By increasing the temperature of prestirring, the yield of **4** is decreased. The yield of **1** rises until the operation temperature reaches 60 °C (Table 1, entry 2). Here, the thermal degradation of peroxodicarbonate seems to be favored over lignin oxidation.

To investigate the influence of the preoxidation temperature further, experiments referring to the dissolving temperature of Kraft lignin were carried out. An attempt was made to rule out the possibility that increased solution temperatures have a significant influence on structural changes in the Kraft lignin. Therefore, samples of Kraft lignin were dissolved in caustic soda at 50 °C. After allowing the samples to cool to 20 °C, peroxodicarbonate was added and the mixture was subjected to heat treatment (Table 1, entry 7). No significant changes in yields could be observed. This suggests that the critical factor is not the dissolution at elevated temperature, but rather the temperature of the preoxidation step with peroxodicarbonate (see Table S6, Supporting Information).

By varying the initial amount of peroxodicarbonate of 3 mmol with ±0.5 mmol, the yields could generally not be improved. By increasing the amount of Kraft lignin from 0.18 wt% up to 0.80 wt% all yields of monoaromatics significantly decrease (**Table** **2**, entry 1–4). However, all four monoaromatics could be detected and quantified. It is noteworthy that the amount of 1 compared to the total amount of monoaromatics is decreasing, whereas 3 is increasing. We assumed that the formation of 3 is easier compared to other monoaromatics, while the formation of 1 is getting affected by this. However, 4 is always formed in the same amount independent on the total amount of monoaromatics generated. This indicates that 4 may not only be an overoxidation product but directly formed within the degradation process. It confirms that highly concentrated peroxodicarbonate is more a potent oxidizer and the dominant reaction pathway is the direct formation of vanillic acid from lignin. Neither prolonging nor shortening of the heat treatment was beneficial for the formation of monoaromatics. Although the yield of 1 could not be significantly increased, the total amount of monoaromatics was considerably higher than in the approach by Zirbes et al.^[^ ^52^ ^]^ We decided to carry out further optimization in this direction. Since the greatest influence according to the yield of monoaromatics has been observed during optimization of preoxidation (first step), we focused again on the addition of peroxodicarbonate itself (Table 2 entry 1–4 vs. entry 5–8). Due to the degradation reaction at higher temperatures for peroxodicarbonate, lower temperatures were investigated.

**Table 2:** Oxidative degradation of Lineo Kraft lignin to monoaromatics vanillin (1), acetovanillone (2), guaiacol (3), and vanillic acid (4) using peroxodicarbonate as oxidizer varying the lignin feed concentration.

Entry	Lignin conc. in feed [wt%]	Lignin conc. in reaction mixture [wt%]	Total yield of mono aromaticsa) [wt%]	Ratio of monoaromatics
1	0.20	0.18	11.5	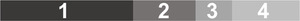
2	0.60	0.48	5.6	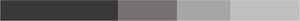
3	0.89	0.66	5.1	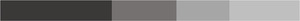
4	1.19	0.80	4.2	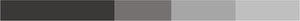
5	0.20	0.18	12.4	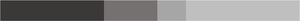
6	0.60	0.48	9.6	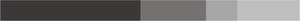
7	0.89	0.66	7.8	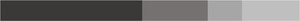
8	1.19	0.80	6.9	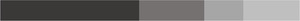

a) The yield was determined by GC‐FID using *n–*dodecylbenzene as internal standard and refers to the amount of lignin employed. Numbers in the graph: 1: Vanillin; 2: Acetovanillone; 3: Guaiacol; 4: Vanillic acid. Reaction conditions: 50 g 3 mol L^−1^ NaOH, single addition of 3 mmol peroxodicarbonate per 100 mg of lignin for entry 1–4, two additions of 1.5 mmol peroxodicarbonate per 100 mg of lignin for entry 5–8, *c*(Peroxodicarbonate) = 800 mmol L^−1^, 30 min pre‐stirring (after each addition), heat treatment temperature: 180 °C, heat treatment time: 8 h.

Additionally, the addition of peroxodicarbonate was divided into several single additions (see Table S10, Supporting Information). It was found that the addition of peroxodicarbonate in two portions at 20 °C prestirring temperature produced comparable total amount of monoaromatics. Unexpectedly, increasing the addition to three portions did not have a favorable effect on the total amount of monoaromatics, which is why further experiments were carried out with two additions. As for the screening with the single addition of peroxodicarbonate, the amount of peroxodicarbonate (see Table S7, Supporting information) and the amount of lignin used were investigated (entry 5–8). The trend for the total amount of monoaromatics was similar to previous data, but it is noteworthy that it was still almost 7 wt% of monoaromatics even with a concentration of 0.80 wt% Kraft lignin. This corresponds to almost a doubling of the total yield of monoaromatics with the same amount of peroxodicarbonate compared to the single addition. Increasing the amount of peroxodicarbonate from 3 to 3.5 mmol L^−1^ 100 mg^−1^ of lignin could increase the total amounts of monoaromatics from 6.9 to 7.8 wt% (see Table S13, Supporting Information).

When comparing the weight‐based concentrations of the total monoaromatic fraction in the reaction mixture, we would like to point out that this is more than twice as high for entry 8 as for entry 4 and can be of decisive importance for a downstream separation. Further comparing the percentage distribution of the individual monomers, it is noticeable that **2** and **3** behave similarly to the single addition of peroxodicarbonate, but the ratio of **1** and **4** changes.

Increasing the amount of lignin while maintaining the same ratio of lignin to peroxodicarbonate leads to an increase in the amount of **1** but decreases the amount of **4**. This indicates that **4** is at least partially present as an overoxidation product of **1**. However, the use of less peroxodicarbonate to prevent this leads to a decreased total amount of mono aromatics. Further investigations of the heat treatment time showed that for the same yield of monoaromatics the heat treatment time could be reduced to 4 h, whereby a heat treatment time of 2 h already enables double‐digit yields (see Table S14, Supporting information). Varying the heat treatment temperature did not improve the yields, regardless of whether it was increased or decreased. However, if the heat treatment temperature was decreased, the responding treatment time was prolonged, the decreasing yields could partially be compensated (see Table S20, Supporting information). Lowering the concentration of caustic soda from 3 to 2 mol L^−1^ not only reduces the cost and environmental impact of a potential industrial process but also results in comparable yields.

**Figure 2:**
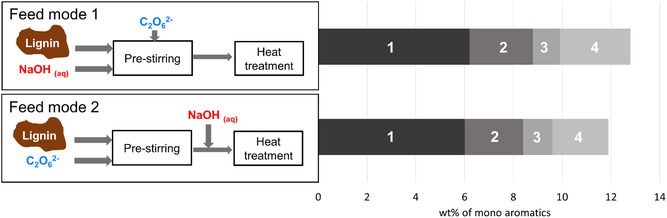
Schematic illustration of the different feed modes. Feed mode 1: dissolution lignin in caustic soda first, subsequent addition of peroxodicarbonate and heat treatment, feed mode 2: addition of peroxodicarbonate directly to solid lignin, subsequent addition of caustic soda and heat treatment. Numbers in the graph: 1: Yield of vanillin; 2: Yield of acetovanillone; 3: Yield of guaiacol; 4: Yield of vanillic acid.

By exchanging the caustic soda with just water the total amount of monoaromatics dropped tremendously. This indicates the crucial role of the solvent but also raised the questions whether a certain basicity of the solvent or a certain ion concentration is necessary, as well as the question in which step of the protocol the caustic soda as an additive plays the important role. Therefore, caustic soda was replaced once with sodium sulfate and once with sodium chloride. For sodium sulfate none of the monomers could be achieved and for sodium chloride a similar low amount of monoaromatics as with water was achieved. We therefore anticipate that the basicity is possibly the more decisive parameter compared to the ion concentration. To investigate in which step of the protocol the addition of caustic soda has the positive impact, we changed the order of operations and defined different “feed modes” (**Figure** **2**). Instead of dissolving lignin in caustic soda first (feed mode 1), peroxodicarbonate was added in two batches to lignin as powder (feed mode 2). When adding caustic soda after the prestirring step followed by the subsequent heat treatment, comparable yields of mono aromatics between the feed modes could be achieved. As in feed mode 2 the lignin did not completely dissolve in the peroxodicarbonate on a laboratory scale, we assumed that this is the reason for the slightly reduced yields. It is noteworthy that the concentration of lignin is around 2.3 wt% in the peroxodicarbonate. This leads to the conclusion that caustic soda is not needed for the preoxidation step itself. Running the reaction in peroxodicarbonate only without the subsequent addition of caustic soda leads to very low yields, comparable with the yields for the feed mode 1 reaction with water. This implies that a certain concentration of caustic soda is required for heat treatment. Experiments replacing the caustic soda with mixed carbonate stock solution used for the peroxodicarbonate generation couldn't achieve the same yields as with caustic soda.

**Figure 3:**
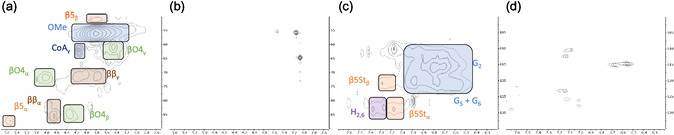
2D HSQC NMR spectra of a) Kraft lignin, aliphatic oxygenated region; b) Kraft lignin after oxidation, aliphatic oxygenated region; c) Kraft lignin, aromatic region; d) Kraft lignin after oxidation, aromatic region.

To investigate the change of the Kraft lignin, 2D heteronuclear single quantum coherence (HSQC) experiment was employed (**Figure** **3**). First, a spectrum of untreated lignin was taken. Like the studies by Zirbes et al. two areas were considered: the aliphatic oxygenated side chain region (δ_C_/δ_H_ 50–90/2.5–5.7) and the aromatic/unsaturated region (δ_C_/δ_H_ 100–135/6.2–7.7). Due to the fact that after workup (acidification) of the heat‐treated residue no precipitation of remaining Kraft lignin was observed, we assume that the polymer is completely degraded or to a large extent chemically modified. By comparing the extractives with untreated Kraft lignin, a clear degradation of the polyaromatic structure is shown. Typical signals for Kraft lignin which could be identified in the untreated sample were not be identified in the treated lignin sample.^[^ ^44^, ^52^ ^]^ Therefore, a degradation of lignin to smaller, water, and ether soluble fragments is indicated.

**Figure 4:**
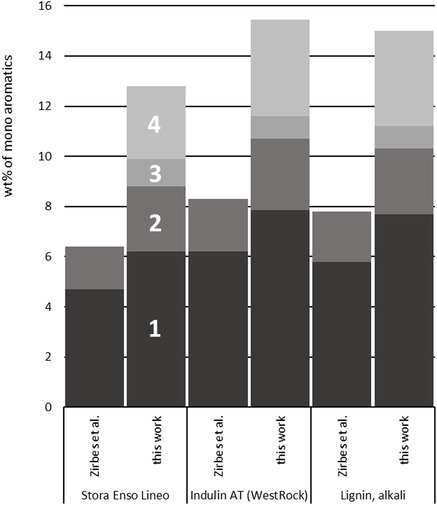
Comparison in the amount of mono aromatics between the method used by Zirbes et al. and this work. Numbers in the Graph: 1: Yield of vanillin; 2: yield of acetovanillone; 3: yield of guaiacol; 4: yield of vanillic acid. The yield was determined by GC‐FID using *n–*dodecylbenzene as internal standard and refers to the amount of lignin employed.

By applying highly concentrated peroxodicarbonate with respect to the newly investigated parameters to further industrial relevant Kraft lignin used by Zirbes et al. the performance of our findings could be validated (**Figure** **4**). Here, a clear increase of the yields for **1** and **2** could be achieved. Additionally, further monoaromatics **3** and **4** could be afforded. The yield of value‐added mono aromatics was almost doubled here. To check the robustness of that method, more industrial relevant Kraft lignins were investigated (see Table S19, Supporting information). In general, yields over 5.6 wt% of **1** could be achieved, next to high yields of **2**, **3**, and **4**. Overall, the total amount of mono aromatics are always double‐digit yields. This proves a stable and robust method for the degradation of Kraft lignin as accessible biomass towards valued added mono aromatics. Further comparing the results of our approach with other works following a single heat treatment process, it has to be noted that the yields we achieve are not only higher, but the process is also faster and results in additional and valuable monomers.^[^ ^34^ ^]^

**Figure 5:**
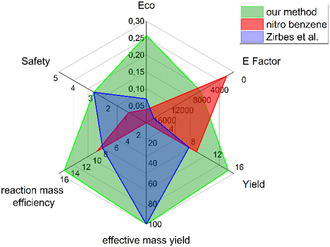
Comparison of our new protocol with the method by Zirbes et al. and the conventional nitro benzene method. For details on calculation of depicted values, see Supporting information, section 6.

The underlying motivation of this work on optimizing numerous approaches for lignin degradation is the development of a sustainable, environmentally friendly technology (**Figure** **5**). Therefore, a quantitative comparison with previous protocols was carried out to put various green chemistry aspects into perspective.^[^ ^59^, ^60^ ^]^ Economic key figures and a safety assessment are also considered. For details on calculation of the depicted values and compared methods, see Supporting Information, Section 6. *Lignin, alkali* by sigma aldrich was selected for the calculations in order to compare the different protocols. A distinct improvement to previous approaches was made, due to the formation of more value‐added monoaromatics in the reaction. Furthermore, we investigated the E factor as important value for the ratio of waste to product and into a safety assessment. The E factor is a simple but important metric to identify the waste to product ratio of a chemical reaction. For our calculations we decided to consider all chemicals which cannot be reused. Therefore, solvents for liquid–liquid extraction are not considered waste, because they can be distilled and reused. It can be shown that our method is in between the conventional approach with nitrobenzene and the method by Zirbes et al.

First, we investigated the costs of the chemical transformation which is an important factor considering an industrial approach. We considered the generated value‐added mono aromatics in contrast to the used lignin including the yields of the protocols (Eco). This is due to the fact that our protocol is using a much smaller volume of peroxodicarbonate solution than by Zirbes et al. but still produces more waste than the nitro benzene approach. Comparing overall yields, our protocol not only achieved additionally **3** and **4**, but also resulted in similar yields of **1** and **2** as in the other protocols. When evaluating the effective mass yield (EMY), which relates the amount of product to the amount of non‐benign reagents required, it becomes also clear that our protocol is significantly more environmentally friendly and less hazardous since our work does not involve toxic reactants like nitro benzene. Additionally, for the nitro benzene workup dichloromethane is used which is carcinogenic. For our work we showed that the ether for liquid–liquid extraction could easily be replaced with ethyl acetate. The CHEM21 selection guide of classical‐ and less classical‐solvents recommends the latter one in terms of Safety, Health and Environment.^[^ ^61^ ^]^ It is not assumed as nonbenign solvent (see Supporting information, Section 6). Furthermore, the reaction mass efficiency (RME) was calculated. This value serves as a measure for the amount of reactant remaining in the product. Our protocol utilizes high‐concentrated peroxodicarbonate, which almost doubles the RME compared to the other approaches. In addition, the product formation comprises 4 monoaromatics in our approach. Finally, we investigated the overall safety of the protocol based on hazards originating from the applied chemical reagents. The protocols including peroxodicarbonate performed significantly better (for details and calculation, see Supporting information, section 6).

## 3 Conclusion

The experiments benchmarked using parameters from Zirbes et al. demonstrated initial yields of 4.7 wt% of **1** and additional 1.7 wt% of the aromatic monomer **2**. Additionally, the high‐concentration peroxodicarbonate protocol of Seitz et al. was reproduced and applied for degradation of lignin using an optimized two‐step approach. The underlying concept lies in the separate prestirring of peroxodicarbonate and the lignin solution and subsequent heat treatment. This has the advantage that the resulting monoaromatics do not oxidize further in an uncontrolled manner, as the oxidizer has already been consumed in the first step. Monoaromatic components were found in total amounts of up to 15.6 wt%. High concentrations of peroxodicarbonate proved more oxidation active, affecting monoaromatic ratios and improving their yields. Moreover, the volume of oxidizer required is diminished when using highly concentrated peroxodicarbonate, which makes it more attractive for industrial applications. Intensive screenings were carried out to optimize both the preoxidation step and the heat treatment. Furthermore, the role of caustic soda was investigated, which showed that the concentration can be lowered to 2 mol L^−1^, which minimizes both costs and the environmental impact. Different feed modes showed caustic soda is essential for the heat treatment step but not the preoxidation step.

Overall, optimizing preoxidation conditions and peroxodicarbonate concentration is crucial for improving mono aromatic yields and composition, offering valuable insights for lignin valorization. The optimized protocol is sustainable and environmentally friendly, achieving high yields without toxic reactants. Economic and safety assessments highlighted its advantages over conventional methods.

## Conflict of Interest

The authors declare no conflicts of interest.

## Supporting information

Supplementary Material

## Data Availability

Data available in article supplementary material.
