# Supplementary material for: Highly Concentrated Peroxodicarbonate for Efficient Oxidative Degradation of Kraft Lignin
Source: ChemSusChem. 2025 Jun 9;18(14):e202500741. doi: 10.1002/cssc.202500741 (PMC12270374; doi:10.1002/cssc.202500741)
Supplement: Supplementary file 1 — Supplementary Material [file CSSC-18-e202500741-s001.pdf]

Supporting Information

Highly Concentrated Peroxodicarbonate for Efficient Oxidative Degradation of Kraft Lignin

Niclas Schupp[a], Therea Rücker[a,b], Elisabeth Glöckner[a], Bernd Wittgens[b] and Siegfried R. Waldvogel*[a,c]

[a]: Department of Electrosynthesis, Max Planck Institute for Chemical Energy Conversion, Stiftstrasse 34-36, 45470 Mülheim an der Ruhr (Germany). E-mail: siegfried.waldvogel@cec.mpg.de

[b]: Department of Process Technology, SINTEF Industry, Richard Birkelands vei 3, 7034 Trondheim (Norway) Process Technology, SINTEF Industry, Trondheim, Trøndelag, NO-7465, Norway

[c]: Karlsruhe Institute of Technology (KIT), Institute of Biological and Chemical Systems - Functional Molecular Systems (IBCS-FMS) Kaiserstraße 12, 76131 Karlsruhe (Germany)

Contents

Electrochemical setup S2
Lignin degradation setup S5
GC Characterization S6
Electrosynthesis of Peroxodicarbonate S11
Oxidative Degradation of Kraft Lignin into mono aromatics S12
Analytics S19
References S24

General remarks

All reagents were used in analytical grades, obtained from common commercial sources, or synthesized following the given literature. The solvents were purified by standard laboratory methods.[1]

Gas chromatography (GC) was performed on a Shimadzu GC-2010 (Shimadzu, Japan) using a HP-5 column (Agilent Technologies, USA; length: 30 m, inner diameter: 0.25 mm, film: 0.25 µm, pre-column: 5 m, carrier gas: hydrogen) with a flame ionization detector (FID) at 310 °C.

NMR spectroscopy: ^{1}H NMR and ^{13}C{^{1}H} NMR spectra were recorded at 25 °C on a Bruker AVANCE III HD 500 MHz NMR spectrometer with a Bruker Prodigy probe (*Bruker BioSpin GmbH*, Rheinstetten, Germany) using DMSO-*d6* as deuterated solvent. The chemical shifts (*δ*) are reported in parts per million (ppm) relative to traces of the residue signal of the corresponding deuterated solvent. Coupling constants *J* are given in Hertz (Hz). Abbreviations used for signal multiplicity: ^{1}H NMR: s = singlet, d = doublet, t = triplet, q = quartet and m = multiplet.

UV/Vis spectroscopy: An Evolution™ 201 UV-Vis-Spectrophotometer (*Thermo Scientific*™, Waltham USA) was used for the determination of the pre-oxidation efficiency. Absorbance spectra were measured between 220 nm and 800 nm with a medium scan speed and 0.5 nm intervals. All spectra were corrected for background intensities by subtracting the spectra of pure solvent measured under identical conditions.

Software: The text was generated using Microsoft Word 2020 (*Microsoft*, Redmond, USA). Figures and schemes were obtained using ChemDraw 22.2.0.3300 *(PerkinElmer Inc.*, Watham, USA) and R Studio 2021.09.2+382 (*Posit PBC*, Boston, USA). NMR and mass spectra were processed using MestReNova v15.0.0-34764 (*Mestrelab Research S.L.*, Santiago de Compostela, Spain).

Electrochemical setup

CONDIAS Flow electrolyzer (Anode surface: 76 cm^2)

The commercial flow eletrolyzer (76 cm^2 anode area) was obtained from *CONDIAS GmbH* (Itzehoe, Germany) and consists of two stainless-steel compartments between which the *DIACHEM*™ BDD anode (Si support, 0.062% B) is located. One stainless steel casing served as cathode, whereas the other functions as cooling device for the back of the anode. An interelectrode gap of 1 mm was achieved via a *Teflon*™ spacer (Figure S2). The cell was connected to a *Ritmo 05* membrane pump (*FINK CHEM+TEC GmbH*, Leinfelden-Echterdingen, Germany) and the cooling device of the cell was attached to a *Julabo F26 cryostat* (*JULABO GmbH*, Seelbach, Germany). Electrolysis was performed with a *TDK Lambda Gen20-76* power source (*TDK- LAMBDA CORPORATION*, Japan) and operated in constant current mode. The electrolysis was performed in cycled-flow mode. Detailed instructions for operating the cell are described in chapter General Protocol A on page S11.

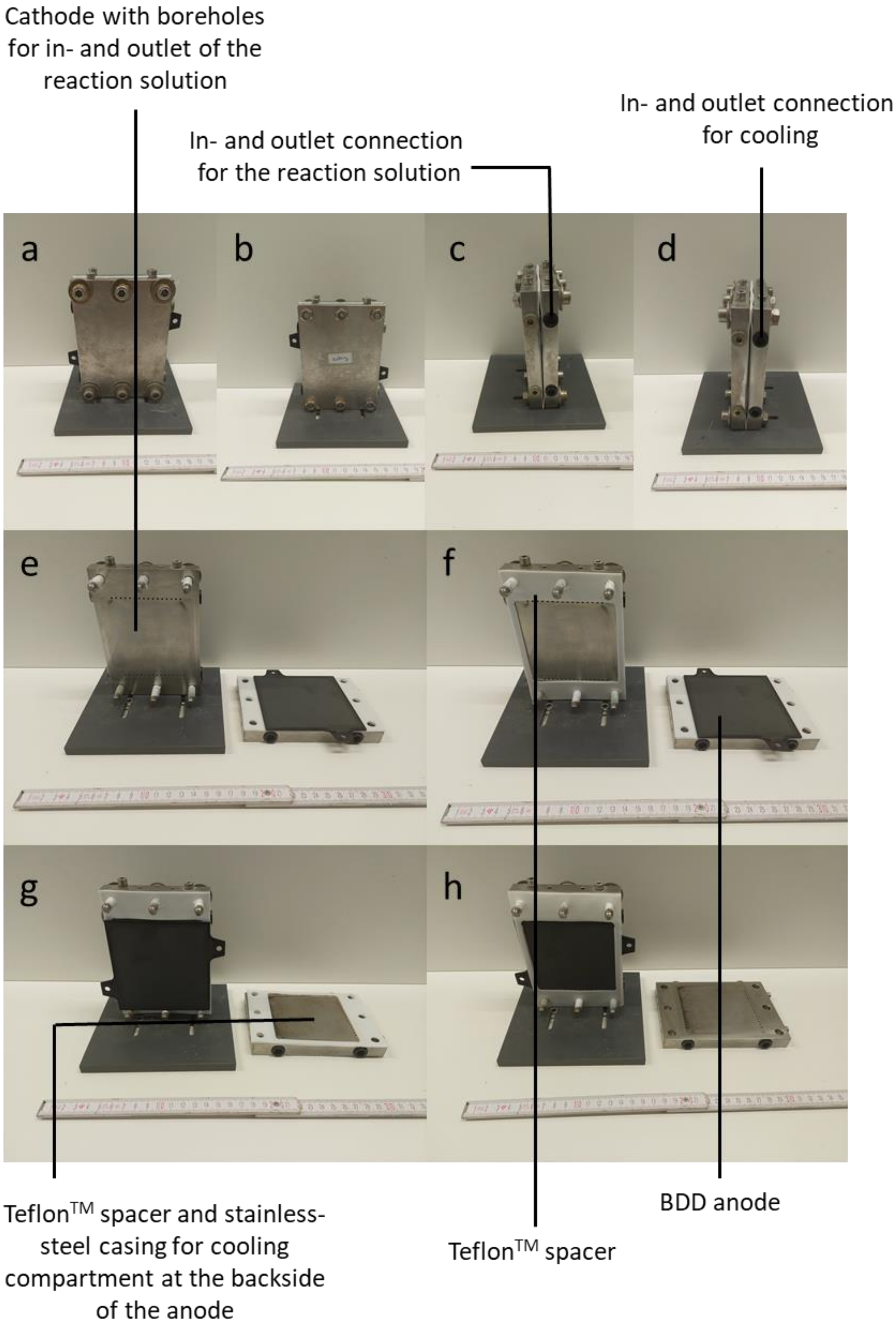

Figure S1:1 *CONDIAS* flow electrolyzer. A–d shows the outside of the cell with the connections for in- and outlet for the reaction solution and cooling. E and f show the BDD anode, as well as the Teflon™ spacer and the cathode. The anode can be contacted from the side and the cathode from the top. The bore holes in the cathode acts as the in- and outlet of the reaction solution in the flow cell. Behind the anode is a second Teflon™ spacer, as well as the second stainless-steel casing attached as shown in g and h. This compartment allows to cool the anode from behind. The cell can be installed with 6 isolated screws und also screwed to a baseplate.

Circular flow cell with cooled copper casing (active anode surface: 3 cm^2)

The electrolysis to peroxodicarbonate was conducted using a previously developed narrow-gap flow electrolyzer.[2] A stainless steel plate served as cathode and a commercial *Diachem*™ boron-doped diamond electrode (2x6 cm, 15 µm BBD on silicon support, *CONDIAS* GmbH, Germany) was used as anode. The interelectrode gap was 0.5 mm. The *Teflon*™ spacer restricted the active anode surface to 3 cm^2. A schematic set-up of the cell is displayed in Figure . Further details can be found in the specified literature.[2] The cell was connected to *Teflon*™ tubing with an inner diameter of 1 mm. The outlet of the tubing was coiled and fixed using zip-ties and immersed in the cooling bath of the cryostat (*RC6* cryostat (*Lauda*, Germany)) together with the electrolyte reservoir (a 100 mL *Schott* flask). The cooling circuit of the cell was connected to the same cryostat using an ethanol/water mixture (1/1, v/v) as cooling fluid at a temperature of 0 °C. The temperature of the electrolyte reservoir was measured using a *DS18B20 temperature* sensor with a USB-connected *cortex-M microcontroller* (*Diamex Produktion und Handel GmbH*, Germany) to allow continuous monitoring. Additionally, the flask was connected to a nitrogen line to remove the hydrogen generated and reduce the risk of ignition. Finally, the electrolyte was pumped using an *Aquamarin 1210 LC PP/EPDM* membrane pump (*Gardner Denver Thomas GmbH*, Germany). The flow rate was adjusted to 100 mL/min *via* the applied potential (10 V at 120–130 mA) from a *Rhode&Schwarz HMP4040* galvanostat and calibrated regularly using a graduated cylinder. The same galvanostat was also used to control the electrolysis in constant current mode. Detailed instructions for operating the cell are described in chapter General Protocol A on page S11.

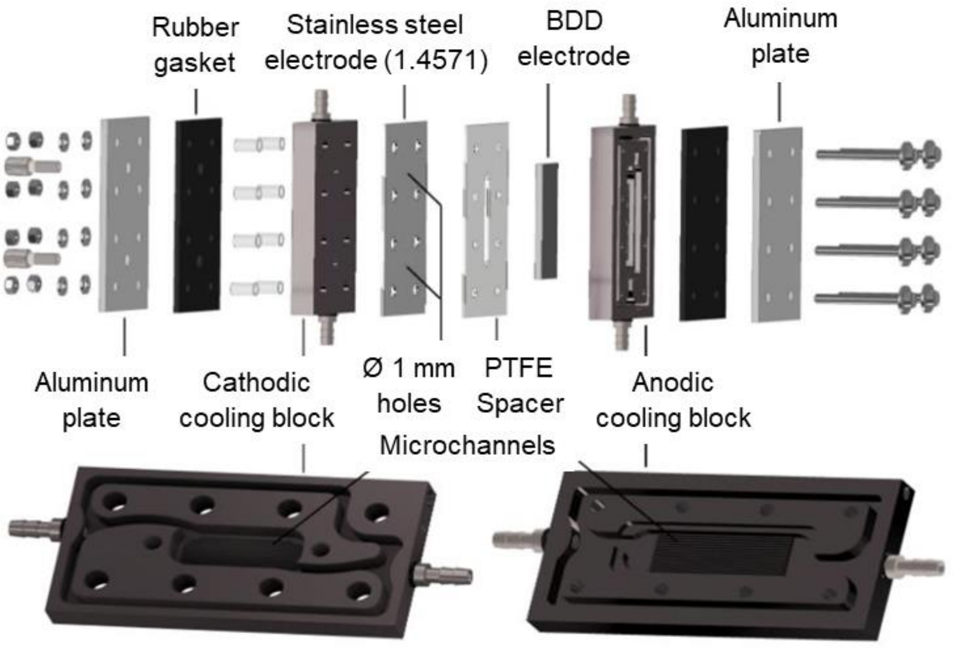

Figure S22: schematic set-up of the flow cell.

Lignin degradation setup

For Kraft lignin degradation a commercially available BR-100 autoclave was obtained from *Berghof* company. The autoclave is made from VA 1.4571 stainless steel with a volume of 170 mL. It is equipped with a thermocouple (thermowell), a rupture disk (Monel, 200 bar) and a pressure gauge. It was heated with an oil bath on a magnetic stir heating plate which was equipped with a thermometer to control the temperature of the oil bath and a timer clock to control the heating time.

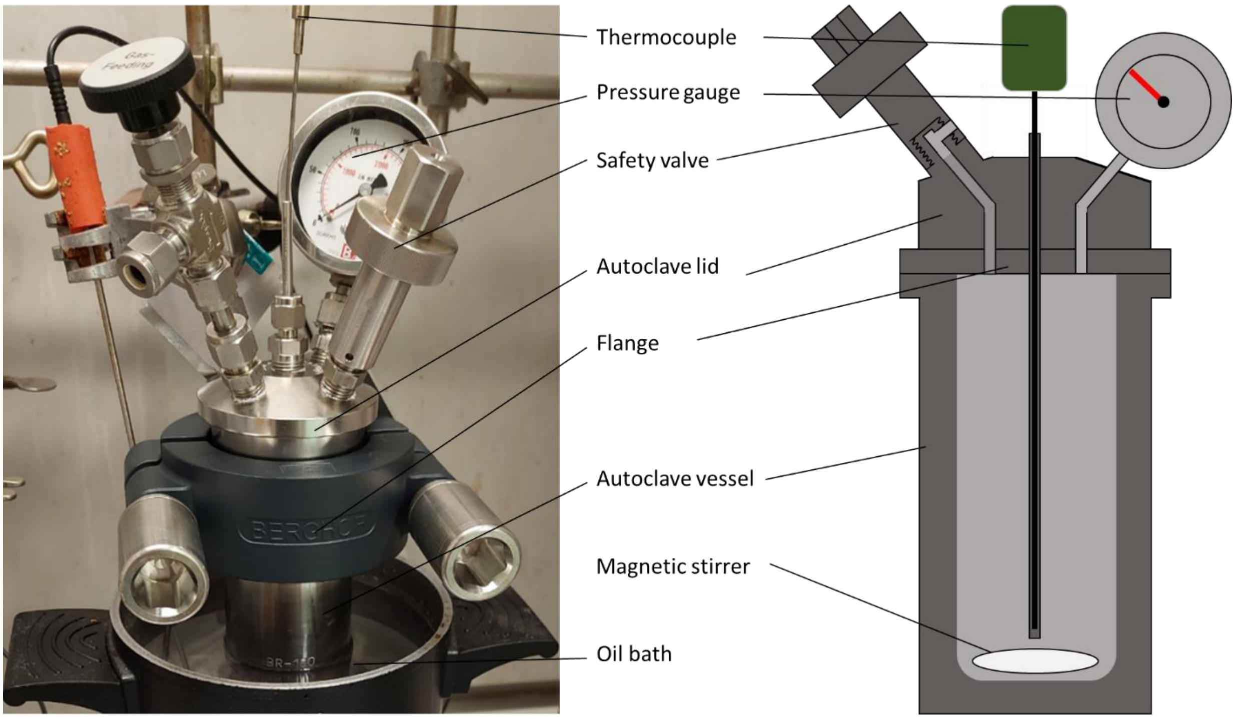

Figure S3: Picture of Autoclave used (left), schematic drawing of autoclave used (right).

GC Characterization

Quantification of vanillin

A precisely defined amount of vanillin **1** was weighed into a GC vial and dissolved in 4 mL ethyl acetate. Afterwards, 2 µL n-dodecylbenzene was added as an internal standard (ISTD) using a 10 µL-Hamilton syringe. Finally, the sample was measured by gas chromatography and the resulting integral ratio between analyte **1** and ISTD was graphically plotted against the corresponding sample weight. For vanillin the corresponding weighed quantities for **1** and the integral ratios obtained are given in Table S1.

Table S1: GC Calibration via internal standard for vanillin **1**.

#	Weight **1** /mg	Integral **1**	Integral ISTD	I (**1**)/ I(ISTD)
1	1.040	52710	225025	0.234
2	2.984	170902	216831	0.788
3	4.970	288113	201213	1.432
4	6.970	445466	217016	2.053
5	8.982	553154	211630	2.614

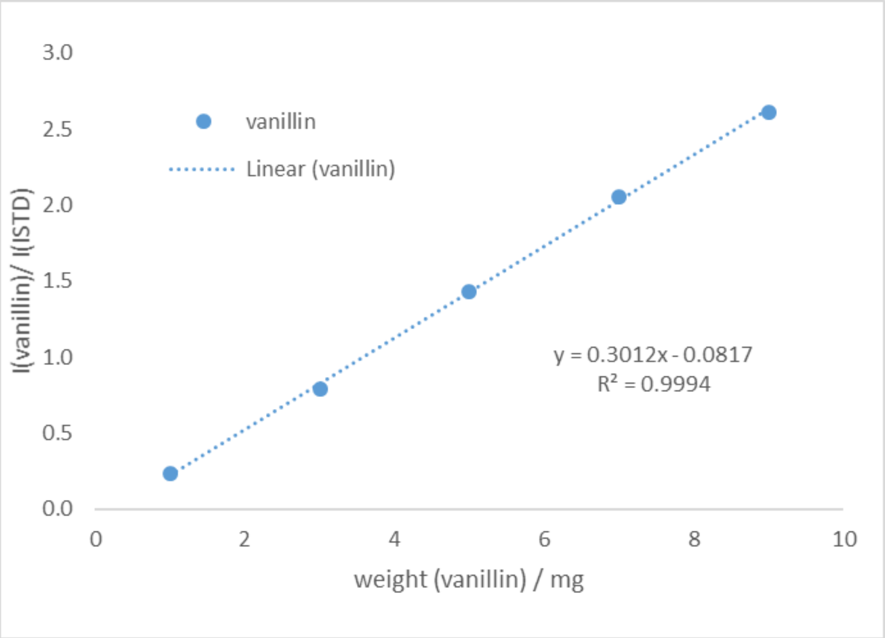

Figure S4: Plot weight vanillin **1** vs. integral ratio vanillin **1** and ISTD with linear fit.

After linear regression the following equation S1 is received:

$$I(\mathbf{1})/I(ISTD) = 0.301 \times m_1 - 0.082 \tag{S1}$$

A calibration with higher concentrations was prepared for analyzing samples from the scale up. A precisely defined amount of vanillin **1** was weighed into a GC vial and dissolved in 4 mL ethyl acetate. Afterwards, 2 µL n-dodecylbenzene was added as an internal standard (ISTD) using a 10 µL-Hamilton syringe. Finally, the sample was measured by gas chromatography and the resulting integral ratio between the analyte **1** and ISTD was graphically plotted against

the corresponding sample weight. For vanillin the corresponding weighed quantities for **1** and the integral ratios obtained are given in Table S2.

Table S2: GC Calibration via internal standard for vanillin **1** for scale up.

#	Weight **1** /mg	Integral **1**	Integral ISTD	I (**1**)/ I(ISTD)
1	11.986	765257	214557	3.567
2	15.025	949301	203577	4-663
3	17.991	1025317	179707	5.705
4	20.989	1424482	224719	6.339

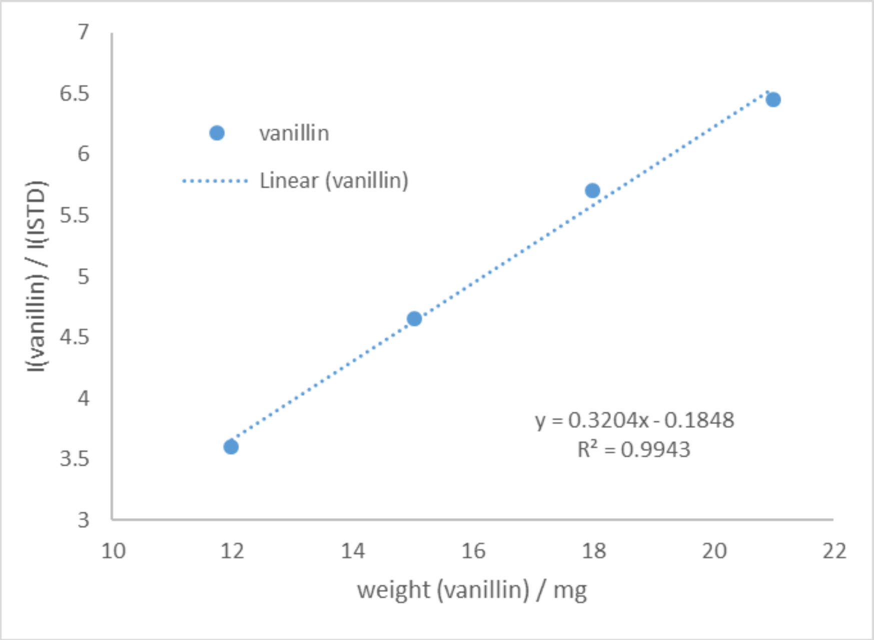

Figure S5: Plot weight vanillin **1** vs. integral ratio vanillin **1** and ISTD with linear fit for scale up.

After linear regression the following equation S2 is received:

$$I(\mathbf{1})/I(ISTD) = 0.320 \times m_1 \; - 0.185 \qquad \text{(S2)}$$

Quantification of acetovanillone

A precisely defined amount of acetovanillone **2** was weighed into a GC vial and dissolved in 4 mL ethyl acetate. Afterwards, 2 µL n-dodecylbenzene was added as an internal standard (ISTD) using a 10 µL-Hamilton syringe. Finally, the sample was measured by gas chromatography and the resulting integral ratio between the analyte **2** and ISTD was graphically plotted against the corresponding sample weight. The corresponding weighed quantities of **2** and the integral ratios obtained are given in Table S3.

Table S3: GC Calibration via internal standard for acetovanillone **2**.

#	Weight **2** /mg	Integral **2**	Integral ISTD	I (**2**)/ I(ISTD)
1	0.985	55972	219187	0.255
2	2.976	190668	215700	0.884
3	4.969	314219	205765	1.527
4	7.017	470874	211289	2.229
5	8.987	608102	215713	2.819
6	11.970	814364	217845	2.513

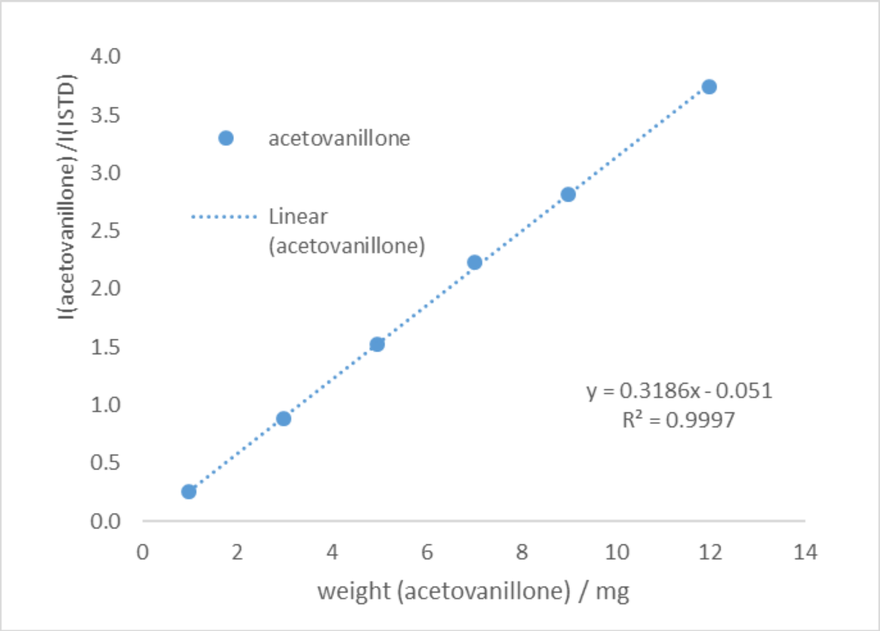

Figure S6: Plot weight acetovanillone **2** vs. integral ratio acetovanillone **2** and ISTD with linear fit.

After linear regression the following equation S3 is received:

$$I(\mathbf{1})/I(ISTD) = 0.319 \times m_1 - 0.051 \qquad \text{(S3)}$$

Quantification of guaiacol

A precisely defined amount of guaiacol **3** was weighed into a GC vial and dissolved in 4 mL ethyl acetate. Afterwards, 2 µL n-dodecylbenzene was added as an internal standard (ISTD) using a 10 µL-Hamilton syringe. Finally, the sample was measured by gas chromatography and the resulting integral ratio between the analyte **3** and ISTD is graphically plotted against the corresponding sample weight. The corresponding weighed quantities of **3** and the integral ratios obtained are given in Table S4.

Table S4: GC Calibration via internal standard for guaiacol **3**.

#	Weight **3** /mg	Integral **3**	Integral ISTD	I (**3**)/ I(ISTD)
1	1.025	55785	192488	0.290
2	2.981	198213	213620	0.928
3	4.967	357330	224364	1.593
4	6.966	499741	215753	2.316
5	9.027	581487	187380	3.103
6	11.982	844331	199500	4.232

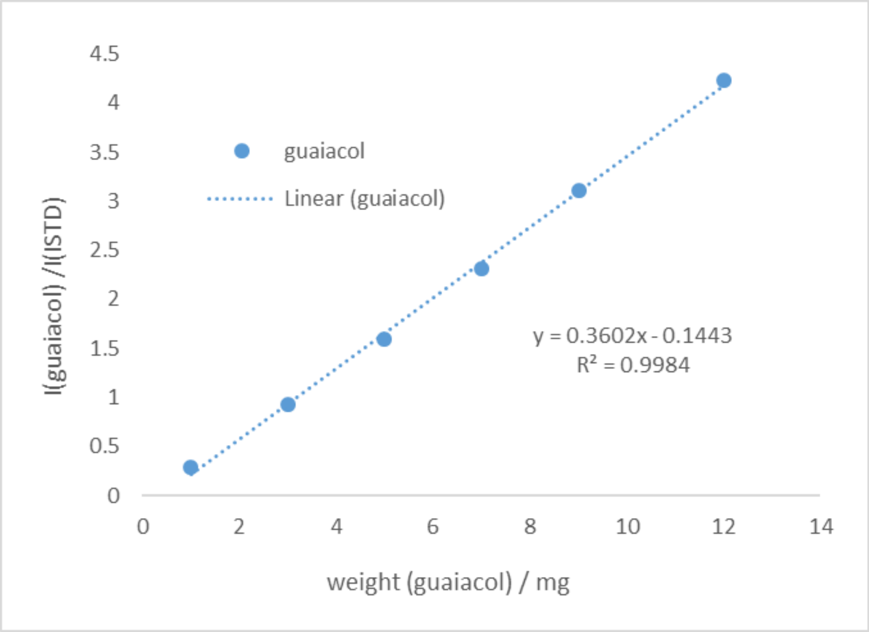

Figure S7: Plot weight guaiacol **3** vs. integral ratio guaiacol **3** and ISTD with linear fit.

After linear regression the following equation S4 is received:

$$I(\mathbf{1})/I(ISTD) = 0.360 \times m_1 - 0.144 \qquad \text{(S4)}$$

Quantification of vanillic acid

A precisely defined amount of vanillic acid **4** was weighed into a GC vial and dissolved in 4 mL ethyl acetate. Afterwards, 2 µL n-dodecylbenzene was added as an internal standard (ISTD) using a 10 µL-Hamilton syringe. Finally, the sample was measured by gas chromatography and the resulting integral ratio between the analyte **4** and ISTD was graphically plotted against the corresponding sample weight. The corresponding weighed quantities of **4** and the integral ratios obtained are given in Table S5.

Table S5: GC Calibration via internal standard for vanillic acid **4**.

#	Weight **4** /mg	Integral **4**	Integral ISTD	I (**4**)/ I(ISTD)
1	0.995	13209	228348	0.058
2	3.028	87952	205865	0.427
3	4.977	168818	203117	0.831
4	7.004	285203	220695	1.292
5	8.997	391571	228433	1.714
6	12.029	529370	210399	2.516

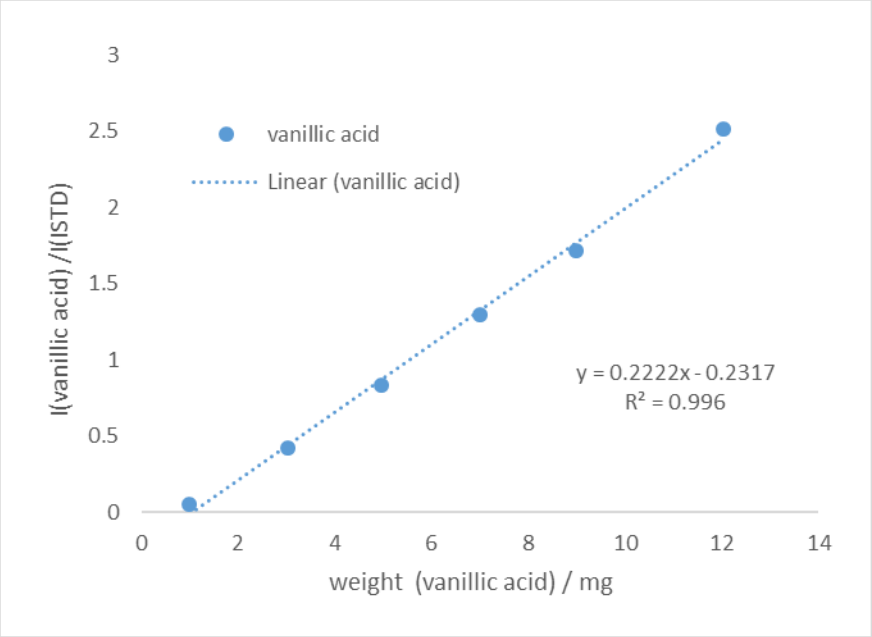

Figure S8: Plot weight vanillic acid **4** vs. integral ratio vanillic acid **4** and ISTD with linear fit.

After linear regression the following equation S5 is received:

$$I(\mathbf{1})/I(ISTD) = 0.222 \times m_1 - 0.232 \quad \text{(S5)}$$

Electrosynthesis of Peroxodicarbonate

General Protocol A for Electrosynthesis of Carbonate Solutions

A1: CONDIAS flow electrolyzer (concentration of peroxodicarbonate: 160–180 mM)

Synthesis of peroxodicarbonate solution:

106.0 g Na_2CO_3 were added into a 1000 mL volumetric flask and filled with water to produce a 1 M Na_2CO_3 electrolyte solution. A volume of 35 mL of this solution was filled into a screw glass container which was placed in an ice bath. The solution was circulated through the electrolysis system at a constant flow rate of 120 mL/min until the temperature kept constant at 5 °C. Then, the solution was electrolyzed using the CONDIAS cell at a constant current density of 260 mA/cm² for 12.83 minutes, corresponding to an applied charge of 15211 (2 F) relative to the carbonate. An interelectrode gap of 1 mm and an anode surface of 76 cm² were used.

Determination of the peroxodicarbonate concentration:

Directly after the electrolysis a 2 mL volumetric pipette was pre-cooled by pumping 2 mL of cold electrolyte solution into the pipette and immediately returning it into the electrolyte three times. Then, up to three 2 mL aliquots of the electrolyte were transferred into Erlenmeyer flasks. The pH was adjusted to 1 by addition of 20% (v/v) H_2SO_4 (6 mL each). To each flask, 5 mL of an aqueous solution of KI (6% in H_2O) and three drops of ammonium heptamolybdate solution (3% in H_2O) were added. The brown solution was titrated against a standard solution of $Na_2S_2O_3$ (0.1 M). The endpoint of the titration was determined by the complete disappearance of the yellow color of free I_2. The resulting average concentration of total oxidizer was then calculated. Concentrations between 160–180 mM of peroxodicarbonate could be achieved.

A2: Circular flow cell with cooled copper casing (concentration of peroxodicarbonate: 800–900 mM)

Synthesis of peroxodicarbonate solution:

95.4 g Na_2CO_3, 155.5 g K_2CO_3 and 22.5 g $KHCO_3$ were added into a 1000 mL volumetric flask and filled with water to produce a 0.9 M Na_2CO_3, 1.125 M K_2CO_3 and 0.225 M $KHCO_3$ electrolyte solution. A volume of 35 mL of this solution was filled into a screw glass container which was placed in the coolant mixture of the cryostat. The solution was circulated through the electrolysis system at a constant flow rate of 120 mL/min until the electrolyte temperature reached steady state at 0 °C. An interelectrode gap of 0.5 mm and an active anode surface of 3 cm² were used. The solution was electrolyzed using the circular flow cell with cooled copper casing at a constant current density of 3.33 A/cm² for 60 minutes, corresponding to an applied charge of 36000 C (4.7 F) relative to the total carbonate. After the electrolysis, the sample container was placed in an ice bath.

Determination of the peroxodicarbonate concentration:

Directly after the electrolysis a 2 mL volumetric pipette was pre-cooled by pumping 2 mL of cold electrolyte solution into the pipette and immediately returning it into the electrolyte three times. Then, up to three 2 mL aliquots of the electrolyte were transferred into Erlenmeyer flasks. The pH was adjusted to 1 by addition of 20% (v/v) H_2SO_4 (6 mL each). To each flask, 5 mL of an aqueous solution of KI (6% in H_2O) and three drops of ammonium heptamolybdate solution (3% in H_2O) were added. The brown solution was titrated against a standard solution of $Na_2S_2O_3$ (0.1 M). The endpoint of the titration was determined by the complete disappearance of the yellow color of free I_2. The resulting average concentration of total

oxidizer was then calculated. Concentrations between 800–900 mM of peroxodicarbonate could be achieved.

Oxidative Degradation of Kraft Lignin into mono aromatics

General Protocol B for the nitro benzene degradation of Kraft lignin

A mass of 100 mg Kraft lignin was placed in a pressure tube and 14 mL of a 2 M sodium hydroxide solution and 0.8 mL of nitro benzene was added. The tube was sealed and placed in an oil bath at 170 °C for 3 h. After cooling the reaction mixture was extracted with dichloromethane (4×50 mL). The aqueous phase was treated with 10 mL of concentrated hydrochloric acid to adjust the pH to 1 and then extracted with ethyl acetate (4×100 mL). The ethyl acetate phase was dried with $MgSO_4$ and the solvent was removed under reduced pressure.

General Protocol C for oxidative Kraft lignin degradation

C1: Feed mode 1

An open autoclave by the *Berghof* company was placed in a water bath for temperature control and pre-tempered for 30 min. Kraft lignin was added and 50 g of a salt solution with a specified concentration was added. The autoclave was covered with a watch glass and pre-stirred for 15 min. Peroxodicarbonate was added and the mixture was pre-stirred before the autoclave was sealed and placed in a pre-heated oil bath at a certain temperature and heated for a defined period. After heat treatment the reactor was cooled to room temperature either in an oil bath or with ice. The reaction mixture was transferred into a 1 L beaker and treated with 50 mL of 50 wt% hydrochloric acid to adjust the pH to 1. The solution was then extracted with either diethyl ether or *tert*-butyl methyl ether (4×100 mL), dried with $MgSO_4$ and the solvent was removed under reduced pressure.

C2: Feed mode 2

A 10 mL glass vial was placed in a water bath for temperature control and pre-tempered for 30 min. Kraft lignin was added and the vial was covered with a watch glass. Furthermore, peroxodicarbonate was added and the mixture was pre-stirred before the vial was transferred to an autoclave (*Berghof* company). 50 g of a 2 M sodium hydroxide solution was used to rinse the vial into the autoclave. Afterwards the autoclave was sealed and placed in a pre-heated oil bath at a certain temperature and heated for a defined period. After heat treatment the reactor was cooled to room temperature either in an oil bath or with ice. The reaction mixture was transferred into a 1 L beaker and treated with 50 mL of 50 wt% hydrochloric acid to adjust the pH to 1. The solution was then extracted with either diethyl ether or *tert*-butyl methyl ether (4×100 mL), dried with $MgSO_4$ and the solvent was removed under reduced pressure.

C3: Feed mode 3

An open autoclave (*Berghof* company) was placed in a water bath for temperature control and pre-tempered for 30 min. 1.74 g Kraft lignin and 30 mL of peroxodicarbonate with a concentration of *c*(peroxodicarbonate) = 850 mmol/L were added, and the mixture was pre-stirred for 30 min, before the autoclave was sealed and placed in a pre-heated oil bath at a 180 °C temperature and heated for 8 h. After heat treatment the reactor was cooled to room temperature in an oil bath. The reaction mixture was transferred into a 1 L beaker and treated with 150 mL of 50 wt% hydrochloric acid to adjust the pH 0 to 1. The solution was then

extracted with either diethyl ether or *tert*-butyl methyl ether (4×100 mL), dried with $MgSO_4$ and the solvent was removed under reduced pressure.

Quantification of Kraft lignin degradation

In general, to determine the mono aromatic yields, the crude product mixture from lignin degradation is resolved in ethyl acetate (4 mL) and 2 µL n-dodecylbenzene was added. Afterwards, the sample is analyzed via GC-FID. Using equations S1 and S2 (for **1**), equation S3 (for **2**), equation S4 (for **3**) and equation S5 (for **4**) yields are provided. The yields are given in wt%, based on the amount of Kraft lignin used.

Table S6: Oxidative degradation of Lineo Kraft lignin to mono aromatics vanillin **1**, acetovanillone **2**, guaiacol **3** and vanillic acid **4** using peroxodicarbonate as oxidizer at different pre-stirring temperatures.

Entry	Pre-stirring temperature	Yield **1**[a]	Yield **2**[a]	Yield **3**[a]	Yield **4**[a]	Total amount of mono aromatics[a]
1	70 °C	4.1 wt%	1.9 wt%	1.2 wt%	2.3 wt%	9.5 wt%
2	60 °C	5.1 wt%	2.4 wt%	1.4 w%	2.6 wt%	11.5 wt%
3	50 °C	4.6 wt%	2.2 wt%	1.2 wt%	4.6 wt%	12.5 wt%
4	40 °C	3.9 wt%	2.1 wt%	1.1 wt%	4.9 wt%	12.0 wt%
5	30 C°	3.1 wt%	1.9 wt%	1.3 wt%	5.0 wt%	11.3 wt%
6	20 °C	2.7 wt%	1.6 wt%	1.5 wt%	4.9 wt%	10.7 wt%
7[b]	20 °C	2.8 wt%	1.7 wt%	0.9 wt%	4.9 wt%	10.3 wt%

Reaction conditions: 100 mg Kraft lignin, 50 g 3 M NaOH, 3 mmol peroxodicarbonate, *c*(Peroxodicarbonate) = 800 mmol, pre-stirring time: 30 min, heat treatment temperature: 180 °C, heat treatment time: 8 h. [a]: The yield was determined by GC-FID using *n*-dodecylbenzene as internal standard and refers to the amount of lignin employed. [b] Lignin was dissolved at 50 °C, then cooled down to 20 °C before adding peroxodicarbonate.

Table S7: Oxidative degradation of Lineo Kraft lignin to mono aromatics vanillin **1**, acetovanillone **2**, guaiacol **3** and vanillic acid **4** using peroxodicarbonate as oxidizer in different amounts per 100 mg Lignin.

Entry	Amount of Peroxodicarbonate	Yield **1**[a]	Yield **2**[a]	Yield **3**[a]	Yield **4**[a]	Total amount of mono aromatics[a]
1	2.0 mmol	4.5 wt%	2.0 wt%	1.5 wt%	2.4 wt%	10.4 wt%
2	2.5 mmol	5.0 wt%	2.2 wt%	1.2 wt%	2.4 wt%	10.8 wt%
3	3.0 mmol	5.1 wt%	2.4 wt%	1.4 wt%	2.6 wt%	11.5 wt%
4	3.5 mmol	4.6 wt%	2.2 wt%	1.2 wt%	2.5 wt%	10.5 wt%

Reaction conditions: 100 mg Kraft lignin, 50 g 3 M NaOH, *c*(Peroxodicarbonate) = 800 mmol, pre-stirring temperature: 60 °C, pre-stirring time: 30 min, heat treatment temperature: 180 °C, heat treatment time: 8 h. [a]: The yield was determined by GC-FID using *n*-dodecylbenzene as internal standard and refers to the amount of lignin employed.

Table S8: Table 2: Oxidative degradation of Lineo Kraft lignin to mono aromatics vanillin **1**, acetovanillone **2**, guaiacol **3** and vanillic acid **4** using peroxodicarbonate as oxidizer with different weight percentage of Kraft lignin according to the total reaction volume.

Entry	Amount of Lignin in reaction volume	Yield **1**[a]	Yield **2**[a]	Yield **3**[a]	Yield **4**[a]	Total amount of mono aromatics[a]
1	0.18 wt%	5.1 wt%	2.4 wt%	1.4 wt%	2.6 wt%	11.5 wt%
2	0.48 wt%	2.2 wt%	1.1 wt%	1.0 wt%	1.3 wt%	5.6 wt%
3	0.66 wt%	1.9 wt%	1.1 wt%	0.9 wt%	1.2 wt%	5.1 wt%
4	0.80 wt%	1.6 wt%	0.8 wt%	0.9 wt%	0.9 wt%	4.2 wt%

Reaction conditions: 50 g 3 M NaOH, 3 mmol peroxodicarbonate per 100 mg of lignin, *c*(Peroxodicarbonate) = 800 mmol, pre-stirring temperature: 60 °C, pre-stirring time: 30 min, heat treatment temperature: 180 °C, heat treatment time: 8 h. [a]: The yield was determined by GC-FID using *n*-dodecylbenzene as internal standard and refers to the amount of lignin employed.

Table S9: Oxidative degradation of Lineo Kraft lignin to mono aromatics vanillin **1**, acetovanillone **2**, guaiacol **3** and vanillic acid **4** using peroxodicarbonate as oxidizer with different heat-treatment times.

Entry	Heat-treatment time	Yield **1**[a]	Yield **2**[a]	Yield **3**[a]	Yield **4**[a]	Total amount of mono aromatics[a]
1	2 h	3.7 wt%	1.2 wt%	0.9 wt%	1.7 wt%	7.5 wt%
2	4 h	4.6 wt%	1.8 wt%	0.9 wt%	1.8 wt%	9.1 wt%
3	6 h	5.3 wt%	2.1 wt%	1.3 wt%	1.9 wt%	10.6 wt%
4	8 h	5.1 wt%	2.4 wt%	1.4 wt%	2.6 wt%	11.5 wt%
5	10 h	5.4 wt%	2.5 wt%	1.4 wt%	2.2 wt%	11.4 wt%

Reaction conditions: 100 mg Kraft Lignin, 50 g 3 M NaOH, 3 mmol peroxodicarbonate, *c*(Peroxodicarbonate) = 800 mmol, pre-stirring temperature: 60 °C, pre-stirring time: 30 min, heat treatment temperature: 180 °C. [a]: The yield was determined by GC-FID using *n*-dodecylbenzene as internal standard and refers to the amount of lignin employed.

Table S10: Oxidative degradation of Lineo Kraft lignin to mono aromatics vanillin **1**, acetovanillone **2**, guaiacol **3** and vanillic acid **4** using two additions of peroxodicarbonate as oxidizer at different pre-stirring temperatures.

Entry	Pre-stirring temperature	Yield **1**[a]	Yield **2**[a]	Yield **3**[a]	Yield **4**[a]	Total amount of mono aromatics[a]
1	0 °C	2.7 wt%	1.6 wt%	0.8 wt%	4.9 wt%	10.0 wt%
2	10 °C	2.6 wt%	1.7 wt%	1.6 wt%	4.9 wt%	10.8 wt%
3[b]	20 °C	4.1 wt%	2.2 wt%	1.2 wt%	3.5 wt%	11.0 wt%
4	20 °C	4.3 wt%	2.2 wt%	1.2 wt%	4.7 wt%	12.4 wt%
5	30 °C	4.6 wt%	2.4 wt%	1.3 wt%	3.6 wt%	11.9 wt%
6	40 °C	4.9 wt%	2.3 wt%	1.3 wt%	2.8 wt%	11.3 wt%
7	50 °C	4.7 wt%	2.2 wt%	1.3 wt%	2.6 wt%	10.8 wt%

Reaction conditions: 100 mg Kraft Lignin, 50 g 3 M NaOH, two additions of 1.5 mmol peroxodicarbonate, c(Peroxodicarbonate) = 800 mmol, pre-stirring time: 30 min after each addition, heat treatment temperature: 180 °C, heat treatment time: 8 h. [a]: The yield was determined by GC-FID using n dodecylbenzene as internal standard and refers to the amount of lignin employed. [b] three additions of peroxodicarbonate.

Table S11 Oxidative degradation of Lineo Kraft lignin to mono aromatics vanillin **1**, acetovanillone **2**, guaiacol **3** and vanillic acid **4** using two additions of peroxodicarbonate as oxidizer in different amounts per 100 mg Lignin.

Entry	Amount of Peroxodicarbonate	Yield **1**[a]	Yield **2**[a]	Yield **3**[a]	Yield **4**[a]	Total amount of mono aromatics[a]
1	2 x 1.00 mmol	4.2 wt%	2.2 wt%	1.3 wt%	2.9 wt%	10.6 wt%
2	2 x 1.25 mmol	4.6 wt%	2.3 wt%	1.2 wt%	3.2 wt%	11.3 wt%
3	2 x 1.50 mmol	4.3 wt%	2.2 wt%	1.2 wt%	4.7 wt%	12.4 wt%
4	2 x 1.75 mmol	3.2 wt%	2.1 wt%	1.1 wt%	4.4 wt%	10.8 wt%

Reaction conditions: 100 mg Kraft Lignin, 50 g 3 M NaOH, two additions of peroxodicarbonate, c(Peroxodicarbonate) = 800 mmol, pre-stirring temperature: 20 °C, pre-stirring time: 30 min after each addition, heat treatment temperature: 180 °C, heat treatment time: 8 h. [a]: The yield was determined by GC-FID using n dodecylbenzene as internal standard and refers to the amount of lignin employed.

Table S12: Oxidative degradation of Lineo Kraft lignin to mono aromatics vanillin **1**, acetovanillone **2**, guaiacol **3** and vanillic acid **4** using two additions of peroxodicarbonate as oxidizer with different weight percentage of Kraft lignin according to the total reaction volume.

Entry	Amount of Lignin in reaction volume	Yield **1**[a]	Yield **2**[a]	Yield **3**[a]	Yield **4**[a]	Total amount of mono aromatics[a]
1	0.18 wt%	4.3 wt%	2.2 wt%	1.2 wt%	4.7 wt%	12.4 wt%
2	0.48 wt%	4.5 wt%	2.1 wt%	1.0 wt%	2.0 wt%	9.6 wt%
3	0.66 wt%	3.7 wt%	1.7 wt%	0.9 wt%	1.5 wt%	7.8 wt%
4	0.80 wt%	3.2 wt%	1.5 wt%	0.9 wt%	1.3 wt%	6.9 wt%

Reaction conditions: 50 g 3 M NaOH, two additions of 1.5 mmol peroxodicarbonate, c(Peroxodicarbonate) = 800 mmol, pre-stirring temperature: 20 °C, pre-stirring time: 30 min after each addition, heat treatment temperature: 180 °C, heat treatment time: 8 h. [a]: The yield was determined by GC-FID using n dodecylbenzene as internal standard and refers to the amount of lignin employed.

Table S13: Oxidative degradation of Lineo Kraft lignin to mono aromatics vanillin **1**, acetovanillone **2**, guaiacol **3** and vanillic acid **4** using two additions of peroxodicarbonate as oxidizer in different amounts per 600 mg Lignin.

Entry	Amount of Peroxodicarbonate	Yield **1**[a]	Yield **2**[a]	Yield **3**[a]	Yield **4**[a]	Total amount of mono aromatics[a]
1	2 x 1.50 mmol	3.2 wt%	1.5 wt%	0.9 wt%	1.3 wt%	6.9 wt%
2	2 x 1.75 mmol	3.8 wt%	1.7 wt%	0.9 wt%	1.4 wt%	7.8 wt%
3	2 x 2.00 mmol	3.5 wt%	1.6 wt%	0.8 wt%	1.4 wt%	7.2 wt%
4	2 x 2.25 mmol	3.3 wt%	1.5 wt%	0.8 wt%	1.6 wt%	7.2 wt%

Reaction conditions: 600 mg Kraft Lignin, 50 g 3 M NaOH, two additions of peroxodicarbonate, c(Peroxodicarbonate) = 800 mmol, pre-stirring temperature: 20 °C, pre-stirring time: 30 min after each addition, heat treatment temperature: 180 °C, heat treatment time: 8 h. [a]: The yield was determined by GC-FID using n dodecylbenzene as internal standard and refers to the amount of lignin employed.

Table S14: Oxidative degradation of Lineo Kraft lignin to mono aromatics vanillin **1**, acetovanillone **2**, guaiacol **3** and vanillic acid **4** using two additions of peroxodicarbonate as oxidizer with different heat-treatment times.

Entry	Heat-treatment time	Yield **1**[a]	Yield **2**[a]	Yield **3**[a]	Yield **4**[a]	Total amount of mono aromatics[a]
1	2 h	4.7 wt%	2.0 wt%	1.0 wt%	2.4 wt%	10.1 wt%
2	3 h	5.6 wt%	2.4 wt%	1.0 wt%	2.7 wt%	11.6 wt%
3	4 h	6.2 wt%	2.6 wt%	1.1 wt%	2.9 wt%	12.7 wt%
4	4.5 h	6.0 wt%	2.5 wt%	1.1 wt%	2.8 wt%	12.4 wt%
5	5 h	6.0 wt%	2.7 wt%	1.2 wt%	3.2 wt%	13.0 wt%
6	6 h	4.9 wt%	2.2 wt%	1.1 wt%	3.5 wt%	11.6 wt%
7	8 h	4.3 wt%	2.2 wt%	1.2 wt%	4.7 wt%	12.4 wt%

Reaction conditions: 100 mg Kraft Lignin, 50 g 3 M NaOH, two additions of 1.5 mmol peroxodicarbonate, c(Peroxodicarbonate) = 800 mmol, pre-stirring temperature: 20 °C, pre-stirring time: 30 min after each addition, heat treatment temperature: 180 °C, [a]: The yield was determined by GC-FID using n dodecylbenzene as internal standard and refers to the amount of lignin employed.

Table S15: Oxidative degradation of Lineo Kraft lignin to mono aromatics vanillin **1**, acetovanillone **2**, guaiacol **3** and vanillic acid **4** using two additions of peroxodicarbonate as oxidizer with different heat-treatment temperatures.

Entry	Heat-treatment temperature	Yield **1**[a]	Yield **2**[a]	Yield **3**[a]	Yield **4**[a]	Total amount of mono aromatics[a]
1	120 °C	3.6 wt%	1.0 wt%	0.7 wt%	2.1 wt%	7.4 wt%
2	140 °C	4.7 wt%	1.5 wt%	0.7 wt%	2.3 wt%	9.1 wt%
3	160 °C	6.0 wt%	2.2 wt%	0.8 wt%	2.8 wt%	11.8 wt%
4	180 °C	6.2 wt%	2.6 wt%	1.1 wt%	2.9 wt%	12.8 wt%
5	200 °C	4.9 wt%	2.6 wt%	1.7 wt%	2.6 wt%	11.8 wt%

Reaction conditions: 100 mg Kraft Lignin, 50 g 3 M NaOH, two additions of 1.5 mmol peroxodicarbonate, c(Peroxodicarbonate) = 800 mmol, pre-stirring temperature: 20 °C, pre-stirring time: 30 min after each addition, heat treatment time: 4 h. [a]: The yield was determined by GC-FID using *n*-dodecylbenzene as internal standard and refers to the amount of lignin employed.

Table S16: Oxidative degradation of Lineo Kraft lignin to mono aromatics vanillin **1**, acetovanillone **2**, guaiacol **3** and vanillic acid **4** using two additions of peroxodicarbonate as oxidizer with different concentrations of caustic soda.

Entry	Concentration of caustic soda	Yield **1**[a]	Yield **2**[a]	Yield **3**[a]	Yield **4**[a]	Total amount of mono aromatics[a]
1[b]	-	0.7 wt%	0.7 wt%	0.9 wt%	1.7 wt%	3.8 wt%
2	0.5	2.1 wt%	1.4 wt%	1.1 wt%	2.2 wt%	6.7 wt%
3	1	5.7 wt%	2.2 wt%	0.9 wt%	2.9 wt%	11.6 wt%
4	1,5	5.8 wt%	2.2 wt%	1.1 wt%	2.8 wt%	11.9 wt%
5	2	6.2 wt%	2.5 wt%	1.1 wt%	3.2 wt%	12.9 wt%
6[c]	2	5.6 wt%	2.0 wt%	1.1 wt%	2.4 wt%	11.1 wt%
7	2,5	6.0 wt%	2.5 wt%	1.1 wt%	3.1 wt%	12.7 wt%
8	3	6.2 wt%	2.6 wt%	1.1 wt%	2.9 wt%	12.8 wt%
9	4	4.9 wt%	2.4 wt%	1.0 wt%	2.8 wt%	11.1 wt%

Reaction conditions: 100 mg Kraft Lignin, 50 g NaOH, two additions of 1.5 mmol peroxodicarbonate, c(Peroxodicarbonate) = 800 mmol, pre-stirring temperature: 20 °C, pre-stirring time: 30 min after each addition, heat tr eatment temperature: 180 °C, heat-treatment time: 4 h. [a]: The yield was determined by GC-FID using n dodecylbenzene as internal standard and refers to the amount of lignin employed. [b]: just 50g water instead of caustic soda. [c]: Ethyl acetate was used instead of diethyl ether or *tert*-butyl methyl ether as solvent for liquid-liquid extraction.

Table S17: Oxidative degradation of Lineo Kraft lignin to mono aromatics vanillin **1**, acetovanillone **2**, guaiacol **3** and vanillic acid **4** using two additions of peroxodicarbonate as oxidizer with different salts instead of caustic soda.

Entry	Variation in salts	Yield **1**[a]	Yield **2**[a]	Yield **3**[a]	Yield **4**[a]	Total amount of mono aromatics[a]
1	NaOH	6.2 wt%	2.5 wt%	1.1 wt%	3.2 wt%	12.9 wt%
2	NaCl	0.3 wt%	0.4 wt%	0.6 wt%	0.9 wt%	2.2 wt%
3	Na_2SO_4	0.3 wt%	0.5 wt%	0.7 wt%	1.2 wt%	2.7 wt%
4[b]	Na_2CO_3, $KHCO_3$, K_2CO_3	1.5 wt%	0.8 wt%	0.9 wt%	1.1 wt%	4.3 wt%

Reaction conditions: 100 mg Kraft Lignin, 50 g of 2 M salt solution as solvent, two additions of 1.5 mmol peroxodicarbonate, c(Peroxodicarbonate) = 800 mmol, pre-stirring temperature: 20 °C, pre-stirring time: 30 min after each addition, heat treatment temperature: 180 °C, heat-treatment time: 4 h. [a]: The yield was determined by GC-FID using n dodecylbenzene as internal standard and refers to the amount of lignin employed. [b]: A 0.90 M Na_2CO_3, 1.125 M K_2CO_3 and 0.225 M $KHCO_3$ solution was used as solvent.

Table S18: Oxidative degradation of Lineo Kraft lignin to mono aromatics vanillin **1**, acetovanillone **2**, guaiacol **3** and vanillic acid **4** using different feet modes

Entry	Feed mode	Yield **1**[a]	Yield **2**[a]	Yield **3**[a]	Yield **4**[a]	Total amount of mono aromatics[a]
1	1	6.2 wt%	2.6 wt%	1.1 wt%	2.9 wt%	12.8 wt%
2	2	6.0 wt%	2.4 wt%	1.2 wt%	2.3 wt%	11.9 wt%
3	3	0.6 wt%	0.2 wt%	0.2 wt%	0.1 wt%	1.1 wt%

Reaction conditions: 100 mg Kraft Lignin, 50 g 2 M NaOH, two additions of 1.5 mmol peroxodicarbonate, c(Peroxodicarbonate) = 800 mmol, pre-stirring temperature: 20 °C, pre-stirring time: 30 min after each addition, heat treatment temperature: 180 °C, heat-treatment time: 4 h. [a]: The yield was determined by GC-FID using n dodecylbenzene as internal standard and refers to the amount of lignin employed.

Table S19 Oxidative degradation of different Kraft lignin to mono aromatics vanillin **1**, acetovanillone **2**, guaiacol **3** and vanillic acid **4**.

Entry	Kraft lignin	Yield **1**[a]	Yield **2**[a]	Yield **3**[a]	Yield **4**[a]	Total amount of mono aromatics[a]
1	Stora Enso Lineo	6.2 wt%	2.6 wt%	1.1 wt%	2.9 wt%	12.8 wt%
2	Indulin AT	7.9 wt%	2.9 wt%	0.9 wt%	3.9 wt%	15.5 wt%
3	Aldrich, alkali	7.7 wt%	2.6 wt%	0.9 wt%	3.8 wt%	15.0 wt%
4	BASF Kraftlignin	5.7 wt%	2.5 wt%	1.0 wt%	2.9 wt%	12.1 wt%
5	Mercer	5.6 wt%	2.2 wt%	0.9 wt%	2.8 wt%	11.5 wt%
6	BioPiva 100	5.9 wt%	2.4 wt%	1.0 wt%	2.8 wt%	11.9 wt%
7	BASF Lignin, trocken	6.4 wt%	2.4 wt%	1.0 wt%	3.4 wt%	13.2 wt%

Table S20: Oxidative degradation of different Kraft lignin to mono aromatics vanillin **1**, acetovanillone **2**, guaiacol **3** and vanillic acid **4**.

Entry	Variation in heat-treatment	Yield **1**[a]	Yield **2**[a]	Yield **3**[a]	Yield **4**[a]	Total amount of mono aromatics[a]
1	180 °C, 4 h	6.2 wt%	2.6 wt%	1.1 wt%	2.9 wt%	12.8 wt%
2	160 °C, 4 h	6.0 wt%	2.2 wt%	0.8 wt%	2.8 wt%	11.8 wt%
3	160 °C, 6 h	6.0 wt%	2.2 wt%	0.8 wt%	3.4 wt%	12.4 wt%

Analytics

General Protocol D for the UV/VIS spectroscopy

25 mg of Kraft lignin were dissolved in 12.5 mL of a 3 M caustic soda solution. To this solution either 1 mL of a 0.9 M Na_2CO_3, 1.125 M K_2CO_3 and 0.225 M $KHCO_3$ electrolyte or the corresponding quantity of peroxodicarbonate was added. The samples were stirred for 30 min before diluting 200 µL of the samples with 1800 µL of a 3 M caustic soda solution. This diluted solution (2 mL) is transferred into a 10 mm quartz cuvette (*Helma GmbH*) and analyzed using an Evolution™ 201 UV-Vis-Spectrophotometer. The recorded spectra are normalized to the absorbance at 224 nm.

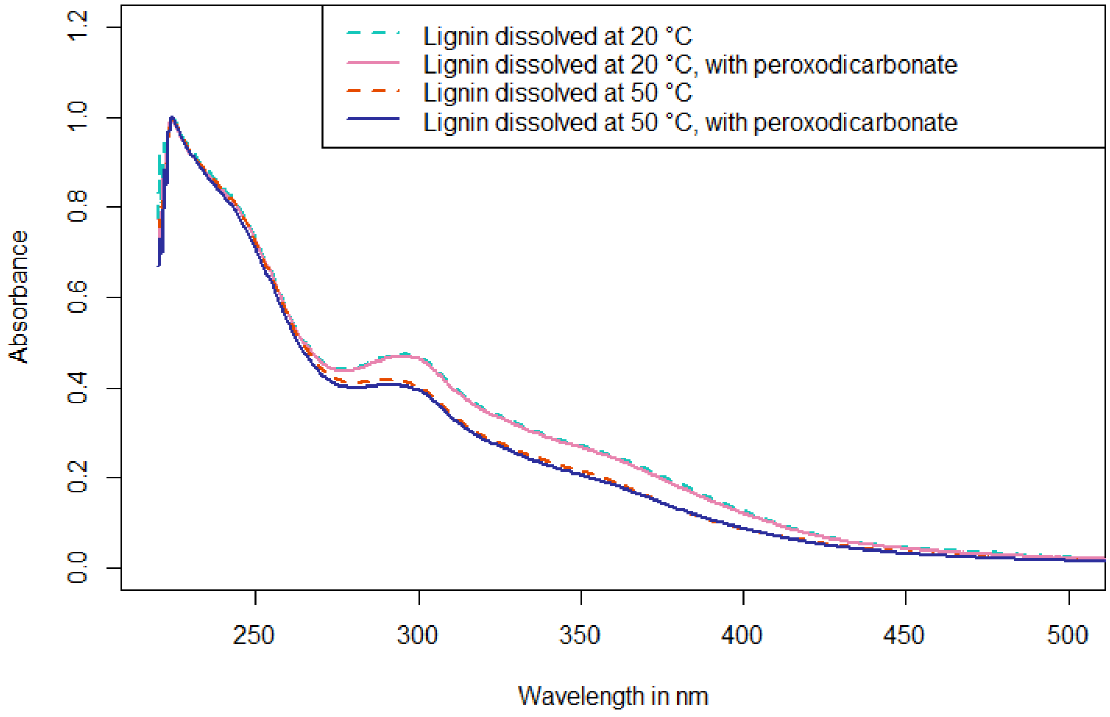

Figure S9: UV/Vis spectra of different samples of Lineo® Kraft lignin before heat treatment.

General Protocol E for the 2D HSQC NMR spectroscopy.

For heteronuclear single quantum coherence (HSQC) NMR spectroscopy of Kraft lignin, around 200 mg of Kraft lignin (*Stora Enso* Lineo®) is dissolved in 0.6 mL DMSO-d6 (*Deutero*) under vigorous stirring. The spectra were acquired at RT on a multi-nuclear resonance spectrometer type Bruker AVANCE III HD 500 MHz NMR spectrometer from Bruker, Karlsruhe, Germany. A hsqcetgpsi2 pulse sequence was used. For the Lignin spectra a matrix of 2048 data points in the ^{1}H-dimension and 256 data points in the ^{13}C-dimension were collected A spectral widths from 17 to -5 ppm (^{1}H-dimension) and 340 to -40 ppm (^{13}C-dimension) were used and the number of scans was 64. For the spectra of the degraded extractives a matrix of 512 data points in the ^{1}H-dimension and 512 data points in the ^{13}C-dimension were collected. A spectral widths from 8 to 0 ppm (^{1}H-dimension) and 200 to 0 ppm (^{13}C-dimension) were used and the number of scans was 32. In both spectra a relaxation delay of 1.5 was used. The spectra were processed with MestreNova. The central solvent peak (DMSO) was used as internal shift reference point (δc = 39.52 ppm, δH = 2.50 ppm). Assignment of the signals was carried out according to literature.[3]

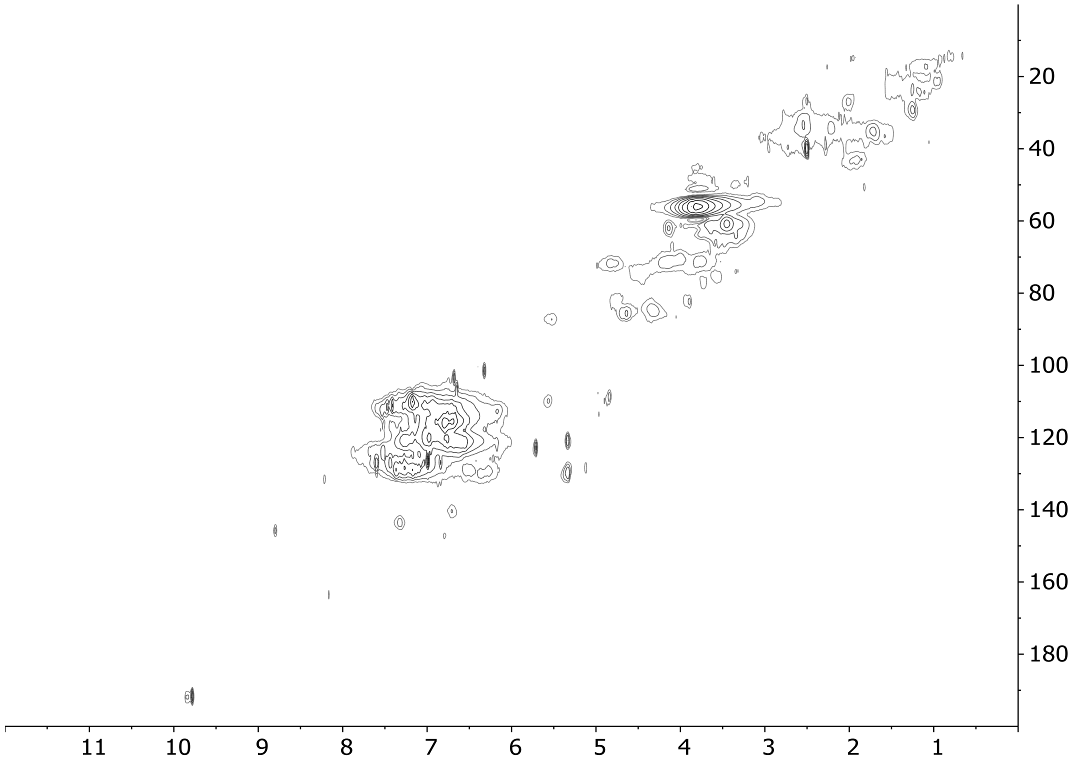

Figure S10: 2D HSQC NMR spectra of Kraft lignin

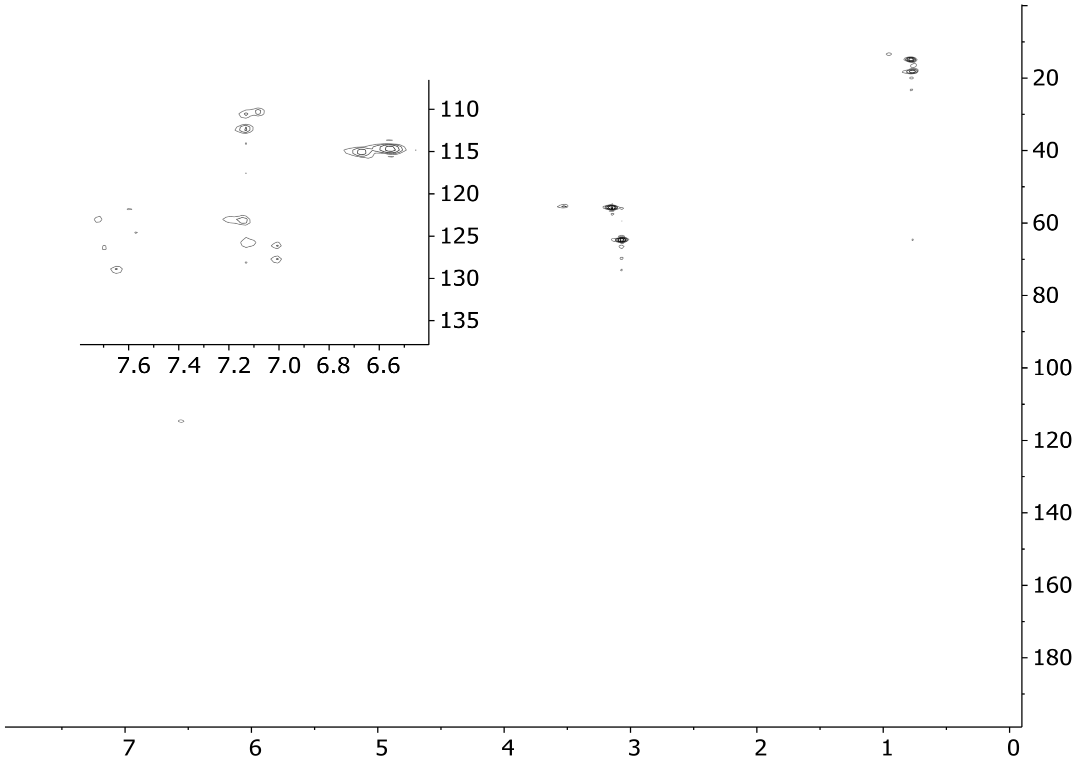

Figure S11: 2D HSQC NMR spectra of Kraft lignin extractives after oxidation

Quantitative comparison with previous protocols

To assess the sustainability of the developed protocol, a comparison was conducted with conventional, published protocols. For a well-rounded evaluation, six different green metrics were analyzed. The comparison focused on the Kraft lignin *Lignin, alkali* by *Sigma Aldrich* and was carried out against the conventional protocol utilizing nitro benzene and the previous published protocol by Zirbes et al. The cost analysis includes only consumed and non-recoverable chemicals, calculated based on a synthesis scale of 1 g of starting material. All prices were sourced from the current Sigma Aldrich catalog for the German market, using the largest available package size to determine the price per gram of chemicals (Status 02.01.2025). Solvents and acids were priced per 2.5 L unless otherwise specified.

The safety assessment was carried out in accordance with the GHS classification of the chemicals present in the protocol. A scale of 1 to 5 was used, where 1 stands for very extremely hazardous and 5 for very safe. The assessment was carried out according to the following table and the safety data sheet of Sigma Aldrich. The smallest GHS rating for a chemical used in the protocol results in the safety of the entire protocol.

GHS rating	Hazard
1	Explosive or oxidizing or toxic or health hazard
2	harmful, flammable, environmental, corrosive (combination of 3 hazards)
3	harmful, flammable, environmental, corrosive (combination of 2 hazards)
4	harmful, flammable, environmental, corrosive (1 hazard)
5	none

ECO was calculated according to the following formula:

$$ECO = \frac{product\ value\ per\ g\ \cdot chemical\ yield\ in\ wt\%}{starting\ material\ cost\ per\ 100g} \quad \text{(S6)}$$

The E-Factor was calculated according to the following formula:[4]

$$E - Factor = \frac{total\ waste\ in\ g}{product\ in\ g} \quad \text{(S7)}$$

Effective mass yield was calculated according to the following formula:[4]

$$EMY = \frac{mass\ of\ disired\ products\ in\ g}{mass\ of\ non - benign\ reagents\ in\ g} \cdot 100\% \quad \text{(S8)}$$

The reaction mass efficiency was calculated according to the following formula:[4]

$$RME = \frac{mass\ of\ product\ in\ g}{mass\ of\ substrate\ in\ g} \cdot 100\% \quad \text{(S9)}$$

Table S21: Specifications for employed chemicals in the protocols.

Substance	CAS	MW g/mol	Price Euro/g	GHS Hazard	GHS Ranking	Specification
Lignin, alkalisch	8068-05-1	10000	0.48	-	5	500 g
Sodium carbonate	497- 19-8	105.99	0.05	GHS07	4	12 kg, powder ≥99.5% ACS reagent
Potassium carbonate	584- 08-7	138.21	0.02	GHS07	4	50 kg, 99% ReagentPlus®
Potassium bicarbonate	298- 14-6	100.12	0.11	-	5	2.5 kg, 99.7% ACS reagent powder, crystals or granules
Sodium hydroxide	1310-73-2	40	0.04	GHS05	4	ACS reagent ≥97.0%, pellets
Vanillin	121- 33-5	152.15	0.20	GHS07	4	2 kg, 99% ReagentPlus®
Acetovanillone	498-02-2	166.17	1.10	GHS07	4	100 g, 98%
Guaiacol	90-05-1	124.14	0.18	GHS07	4	1 L for synthesis
Vanillin acid	121-34-6	168.15	2.04	-	5	50 g for synthesis
Nitro benzene	98-95-3	123.11	0.04	GHS06 GHS08	1	1 L for synthesis
Dichloro-methane	75-09-2	84.93	0.054	GHS 07 GHS08	1	2.5 L, ≥99.8% suitable for HPLC contains amylene as stabilizer
Ethyl acetate	141-78-6	88.11	0.06	GHS07 GHS02	3	2.5 L ACS reagent ≥99.5%
HCl		36.46	0.04	GHS05 GHS07	3	2.5 L, 37% ACS reagent

Our Protocol:

$$ECO = \frac{\left(0.20\frac{€}{g}\cdot 7.7\,wt\%\right)+\left(1.10\,\frac{€}{g}\cdot 2.6\,wt\%\right)+\left(0.18\,\frac{€}{g}\cdot 0.9\,wt\%\right)+\left(2.04\,\frac{€}{g}\cdot 3.8\,wt\%\right)}{0.48\,\frac{€}{g}}$$
$$= 0.26$$

$$E-Factor = \frac{113850\,g}{15\,g} = 7590$$

The EMY metric exceeds 100% for reactions with mainly benign reagents. Since no non-benign reagents are employed effective mass yield was set to be 100%

$$RME = \frac{15\,g}{100\,g}\cdot 100\% = 15\%$$

Protocol by Zirbes et al.:

$$ECO = \frac{\left(0.20\frac{€}{g}\cdot 5.8\,wt\%\right)+\left(1.10\,\frac{€}{g}\cdot 2.0\,wt\%\right)}{0.48\,\frac{€}{g}} = 0.07$$

$$E-Factor = \frac{152600\,g}{7.8\,g} = 152600$$

The EMY metric exceeds 100% for reactions with mainly benign reagents. Since no non-benign reagents are employed effective mass yield was set to be 100%

$$RME = \frac{7.8\,g}{100\,g}\cdot 100\% = 7.8\%$$

Nitro benzene protocol:

$$ECO = \frac{\left(0.20\frac{€}{g}\cdot 9.2\,wt\%\right)}{0.48\,\frac{€}{g}} = 0.04$$

$$E - Factor = \frac{15060\ g}{9.2\ g} = 1637$$

$$EMY = \frac{9.2\ g}{265000960\ g} \cdot 100\% = 3{,}47 \cdot 10^{-6}\%$$

$$RME = \frac{9.2\ g}{100\ g} \cdot 100\% = 9.2\%$$

References

[1] *Purification of Laboratory Chemicals*, Elsevier, **2013**.

[2] A.-K. Seitz, P. J. Kohlpaintner, T. van Lingen, M. Dyga, F. Sprang, M. Zirbes, S. R. Waldvogel, L. J. Gooßen, *Angew. Chem. Int. Ed.* **2022**, *61*, e202117563.

[3] J. Mikkilä, M. Trogen, K. A. Y. Koivu, J. Kontro, J. Kuuskeri, R. Maltari, Z. Dekere, M. Kemell, M. R. Mäkelä, P. A. Nousiainen et al., *ACS Omega* **2020**, *5*, 6130.

[4] D. J. C. Constable, A. D. Curzons, V. L. Cunningham, *Green Chem.* **2002**, *4*, 521.
